# Supplementary figures and images for: TLR2 axis on peripheral blood mononuclear cells regulates inflammatory responses to non-infectious immature dengue virus particles
Source: PLoS Pathog. 2022 Oct 14;18(10):e1010499. doi: 10.1371/journal.ppat.1010499 (PMC9605289; doi:10.1371/journal.ppat.1010499)

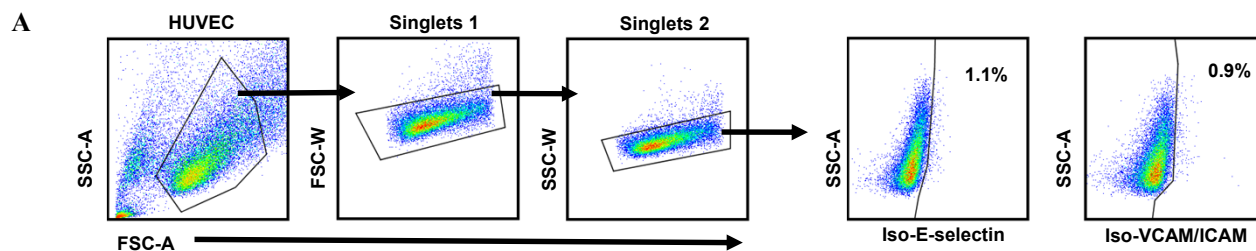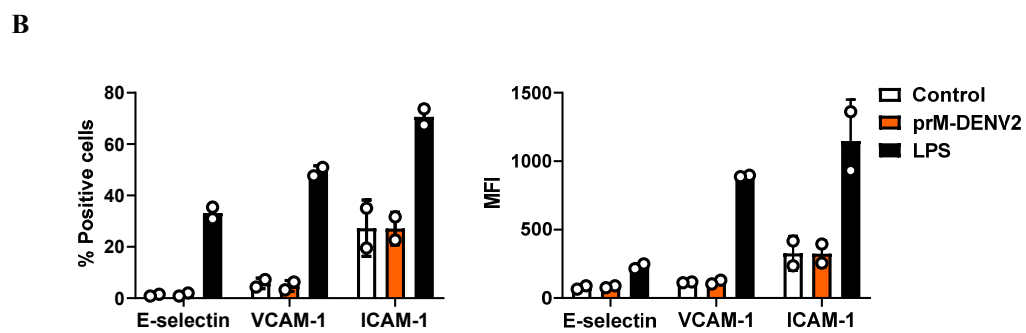

Supplement: S1 Fig — HUVEC were (mock) treated with LPS (1μg/mL) or prM-DENV2 (MOG 300) for 6h. Surface expression of E-selectin, VCAM-1 and ICAM-1 was determined by flow cytometry. (A) Gating strategy on HUVEC to define adhesion molecule positive cells. (B) Expression of adhesion molecules following direct stimuli with prM-DENV2 and LPS. Bar represents mean± SD of two independent biological experiments. (PDF) [file ppat.1010499.s001.pdf]

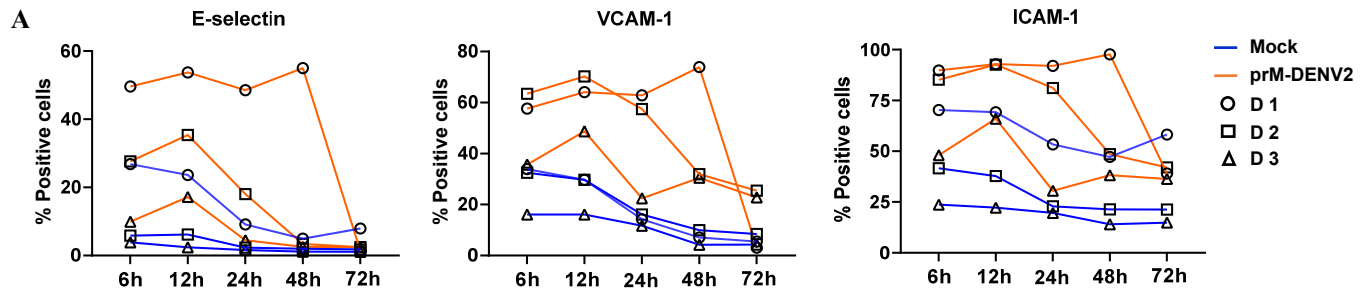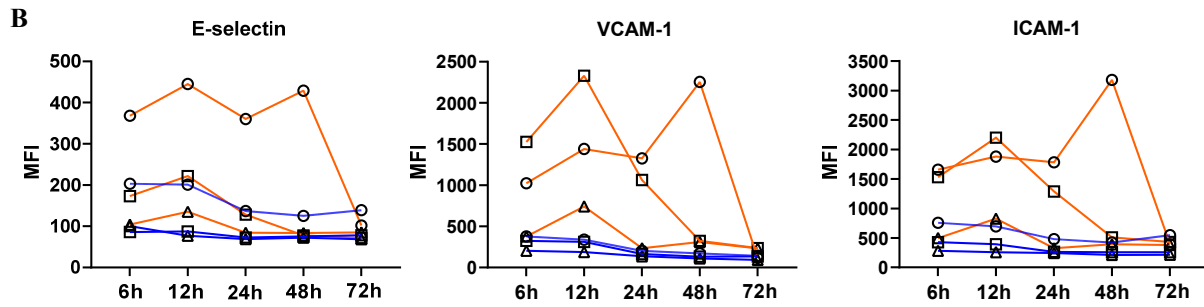

Supplement: S2 Fig — (A) HUVEC were incubated with cell-free supernatants harvested at indicated time points from PBMCs (n = 3, three different donors) exposed to prM-DENV2 at an MOG of 300 or mock treatment. Surface expression of E-selectin, VCAM-1 and ICAM-1 on HUVEC was determined by flow cytometry and represented as (B) percentage of positive cells and (C) MFI. D denotes PBMC donor. (PDF) [file ppat.1010499.s002.pdf]

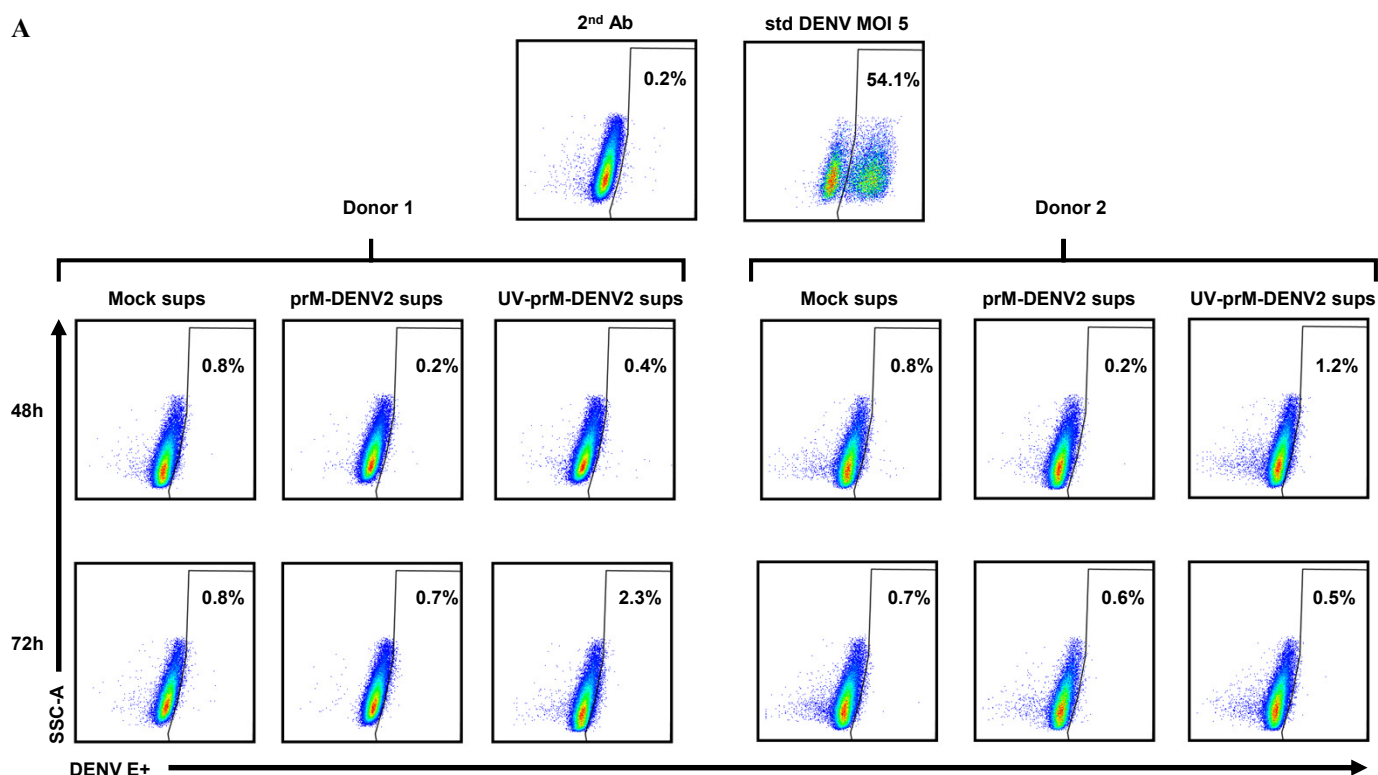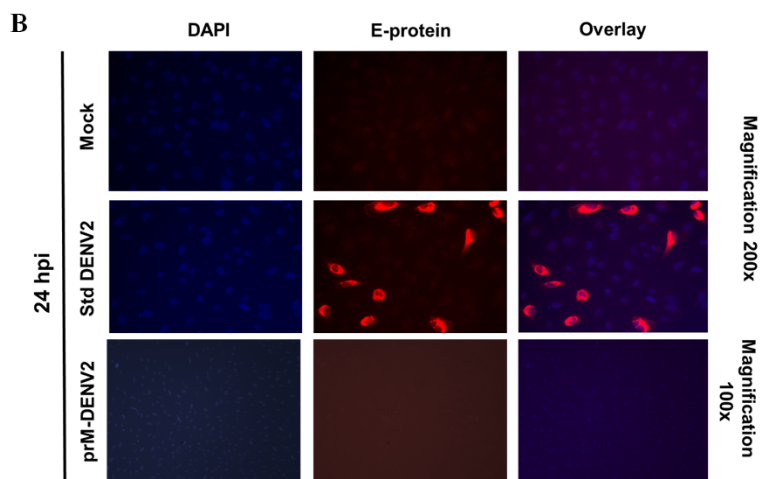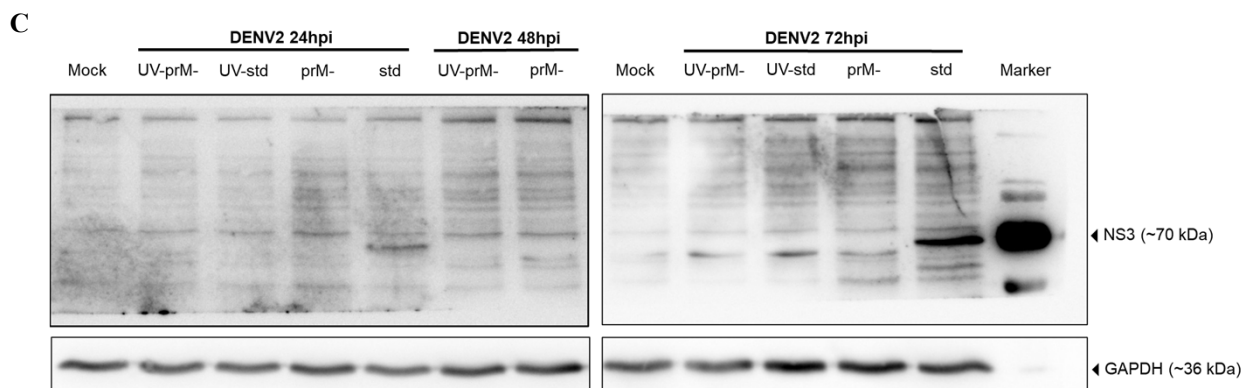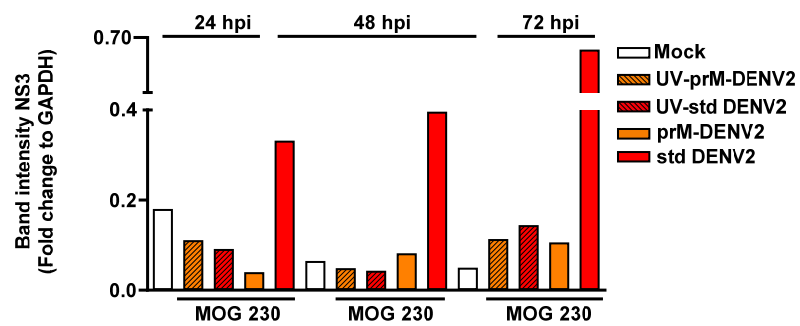

Supplement: S3 Fig — (A) Baby Hamster Kidney cells clone 15 (BHK-15) were incubated for 24h with standard (std) DENV at an MOI of 5 (positive control) or cell-free supernatants from PBMCs (n = 2, two different donors) exposed to prM-DENV2 (MOG 300), UV-prM-DENV2 (MOG 300) or mock treatment for 48h and 72h. % of DENV E+ cells was determined by flow cytometry. (B) HUVEC were infected with Std DENV2 (MOI 20) and prM DENV2 (MOG 10 000) for 24h, cells were then stained for flavivirus E-protein (n = 1). (C) HUVEC were infected with prM-DENV2 and Std DENV2 and their respective UV-inactivated preparations at MOG 230 for 24h, 48h and 72 h. Detection of non-structural protein 3 (NS3) was detected by Western blot. GAPDH was used as a reference. Representative blot and Quantification of NS3 expression shown as fold change to the respective GAPDH control (n = 1). (PDF) [file ppat.1010499.s003.pdf]

A

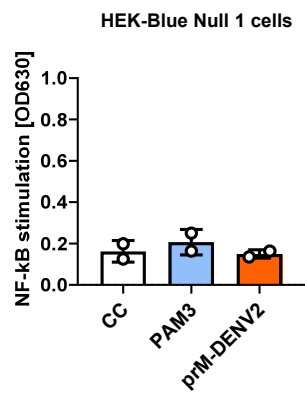

B

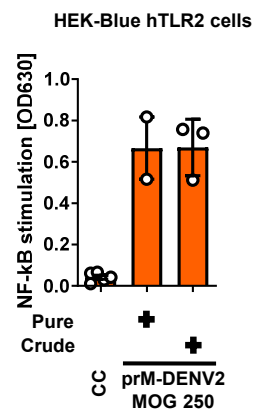

C

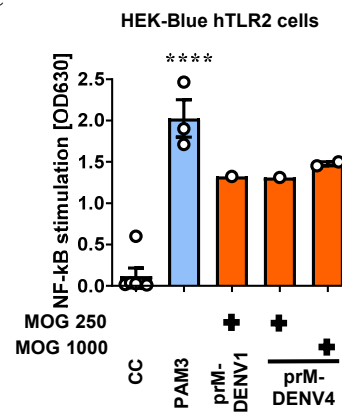

Supplement: S4 Fig — (A) HEK-Blue Null1 cells (mock)-treated with PAM3CSK4 (PAM3, 50 ng/mL, n = 2), prM-DENV2 (MOG 300, n = 2) for 24h. (B) HEK-Blue hTLR2 cells were (mock)-treated with pure (n = 2) or crude (n = 3) preparations of prM-DENV2 (MOG 250) for 24h. (C) HEK-Blue hTLR2 cells (mock)-treated with PAM3CSK4 (PAM3, 50 ng/mL, n = 3), prM-DENV1 (MOG 250, n = 1) and prM-DENV4 (MOG 250, n = 1; MOG 1000, n = 2) for 24h. NF-κB stimulation was assessed by QUANTI-Blue, OD values show the induction of NF-κB. Data represents the mean ± SEM. P values were obtained by one-way ANOVA, Dunnett post hoc test (****P<0.0001). n = independent biological experiments. CC: cellular control. (PDF) [file ppat.1010499.s004.pdf]

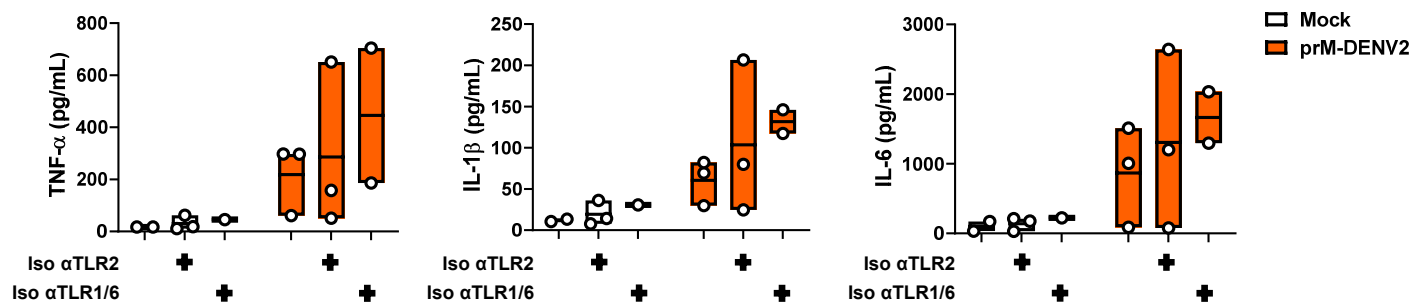

Supplement: S5 Fig — PBMCs from healthy donors (n = 2–3, two to three different donors) were mock-treated with αTLR2 and αTLR1/TLR6 isotype control antibodies (5 μg/mL) for 2 hours prior exposure with prM-DENV2 at MOG 300 for 6h. Cytokine production was measured by flow cytometry using LegendPlex. Each graph shows the production of each cytokine in picograms per milliliter (pg/mL). (PDF) [file ppat.1010499.s005.pdf]

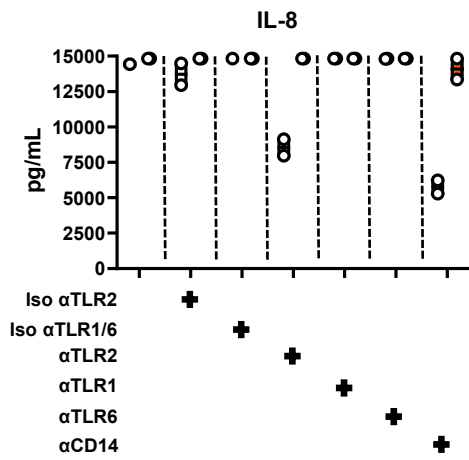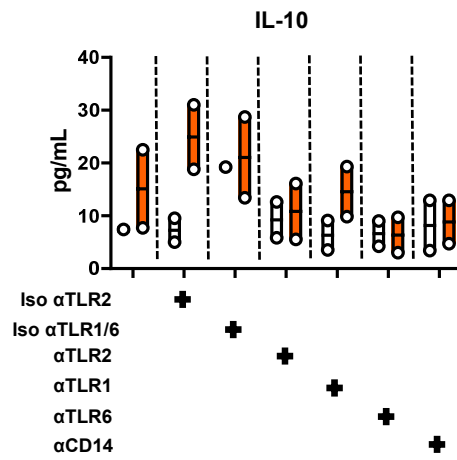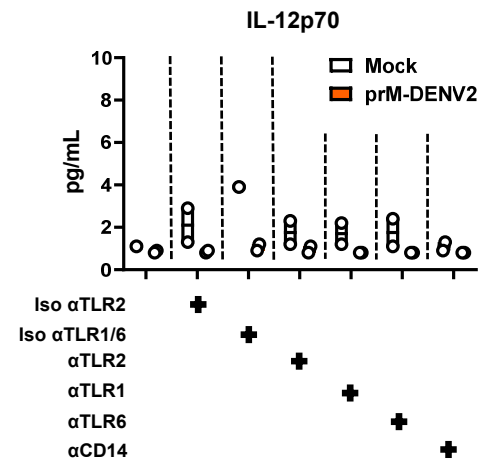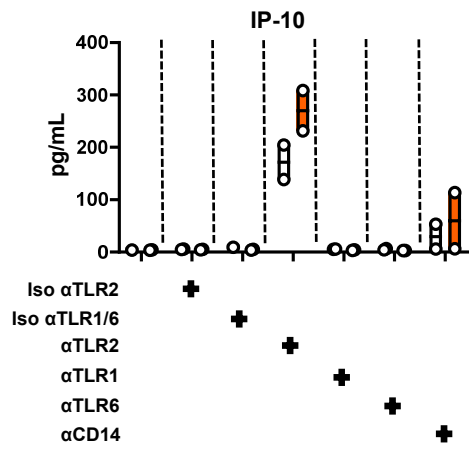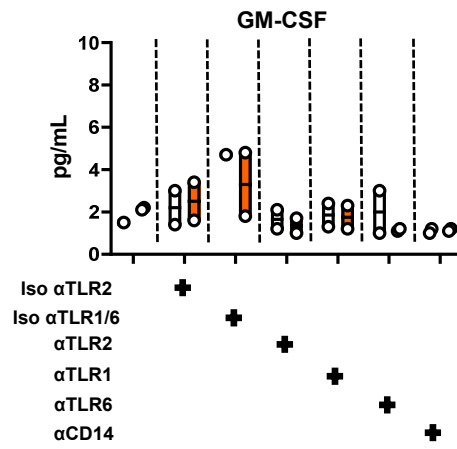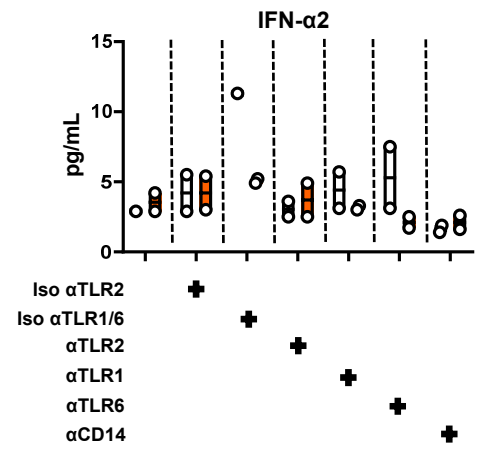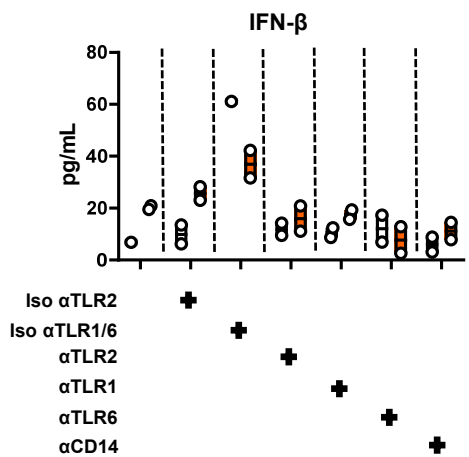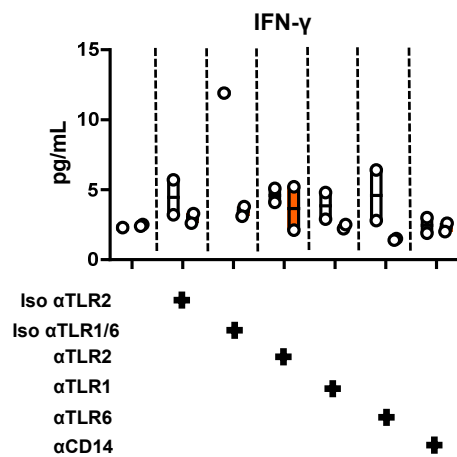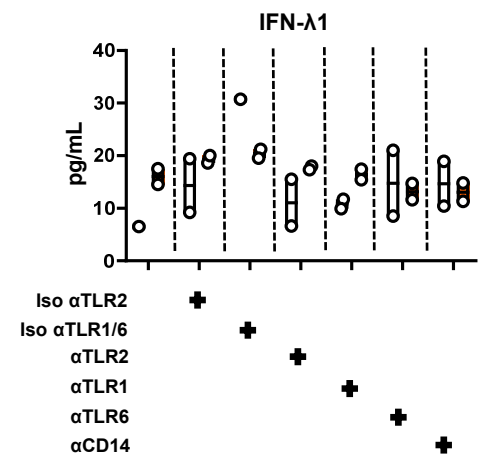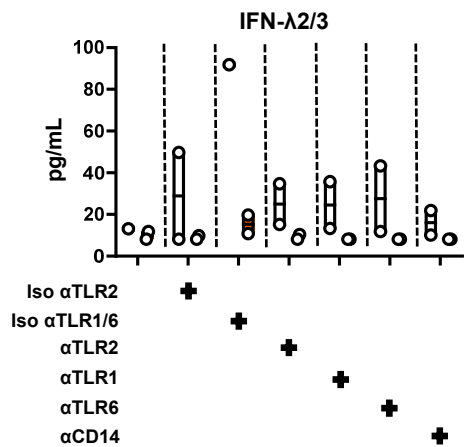

Supplement: S6 Fig — PBMCs from healthy donors (n = 2, two different donors) were mock-treated with αTLR2, αTLR1, αTLR6 (5 μg/mL), αCD14 (3 μg/mL) and isotype control antibodies (5 μg/mL) for 2 hours prior exposure to prM-DENV2 at MOG 300 for 6h. Cytokine production was measured by flow cytometry using LegendPlex. Each graph shows the production of each cytokine in picograms per milliliter (pg/mL). (PDF) [file ppat.1010499.s006.pdf]

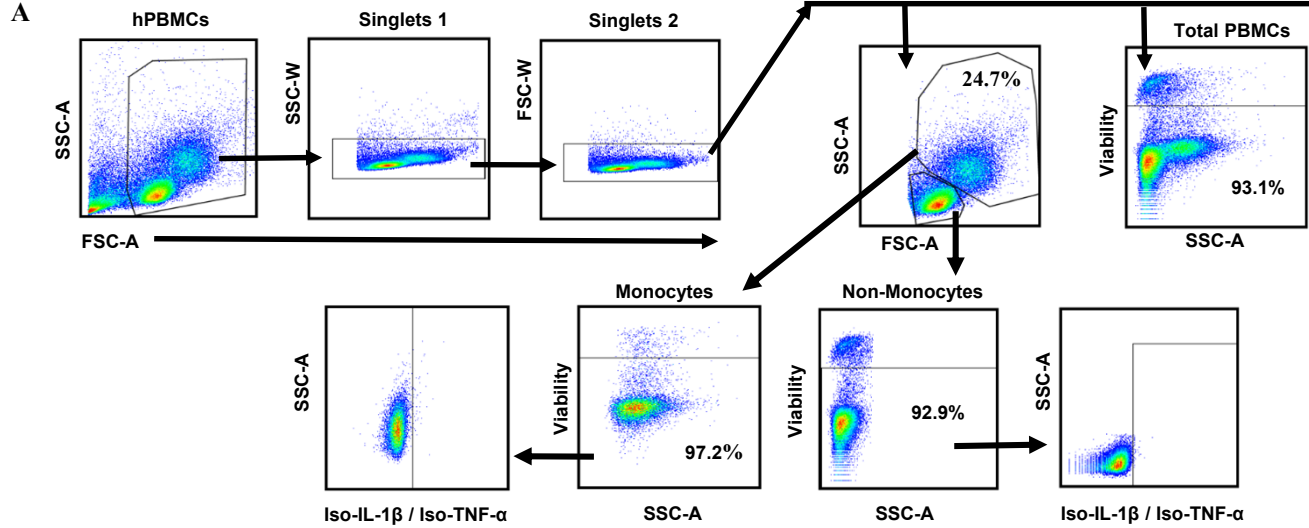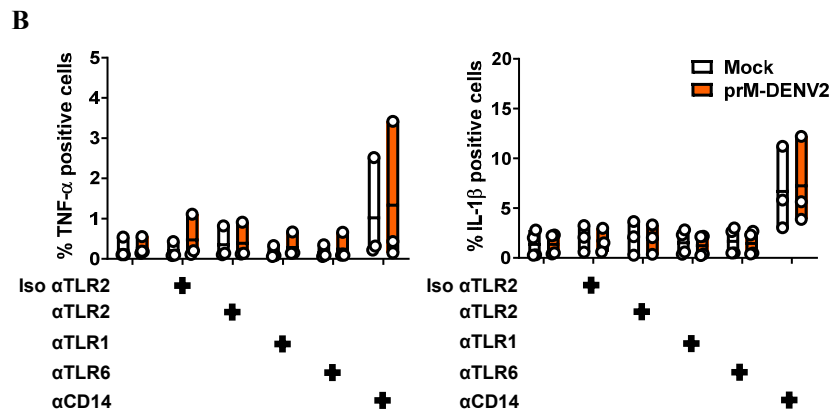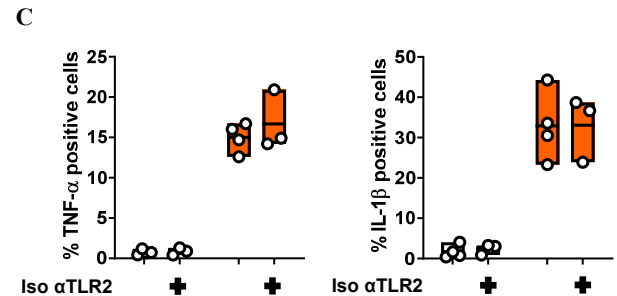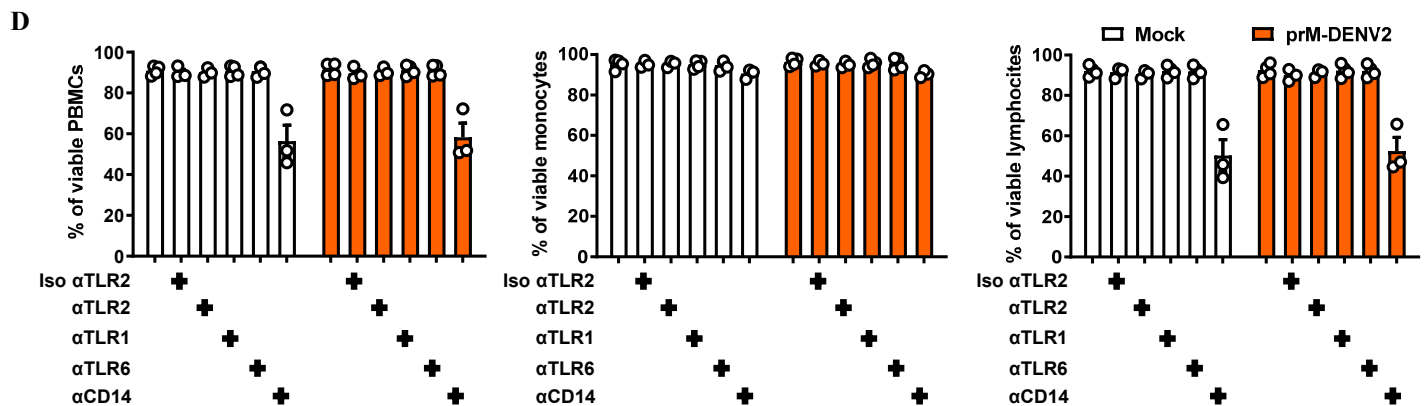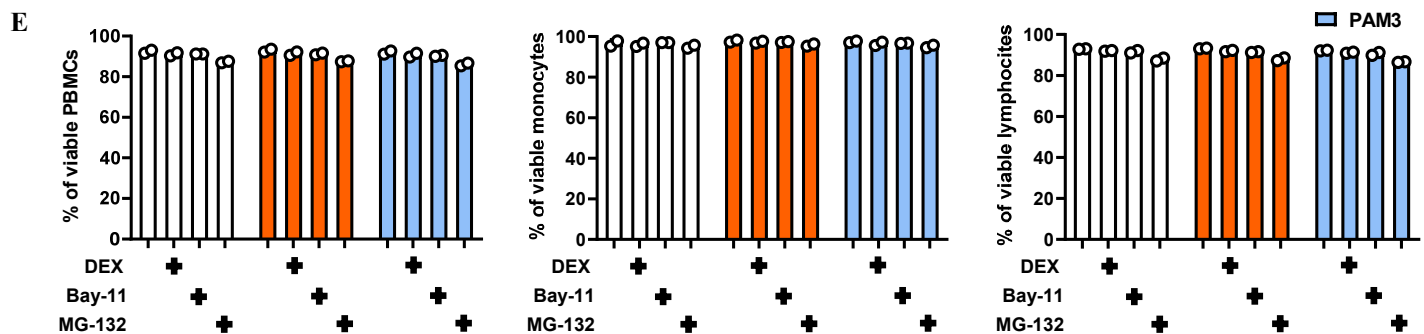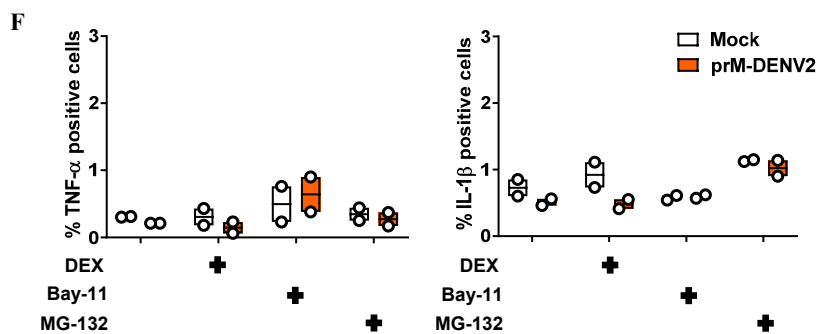

Supplement: S7 Fig — PBMCs from healthy donors (n = 3–5, three to five different donors) were mock-treated with αTLR2 (5 μg/mL), αTLR1 (5 μg/mL), αTLR6 (5 μg/mL), αCD14 (3 μg/mL), αTLR2 and αTLR1/TLR6 isotype control antibodies (5 μg/mL), Dexamethasone (DEX, 10μM), Bay-11 (5μM) and MG-132 (9.5 μg/mL) for 2 hours prior exposure to prM-DENV2 at MOG 300 or PAM3CSK4 (PAM3, 600 ng/mL) for 12h in the presence of Brefeldin-A. (A) Gating strategy to measure the intracellular accumulation of IL-1β and TNF-α in the live monocyte and lymphocyte fractions within the PBMCs. (B, C and F) Intracellular accumulation of IL-1β and TNF-α in live lymphocytes. (D and E) percentage of viable PBMCs, monocytes, and lymphocytes. Bar represents the mean ± SEM. (PDF) [file ppat.1010499.s007.pdf]

**A**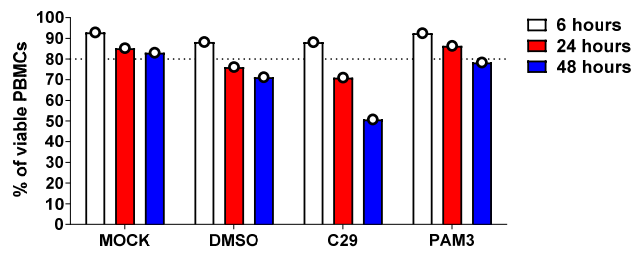**B**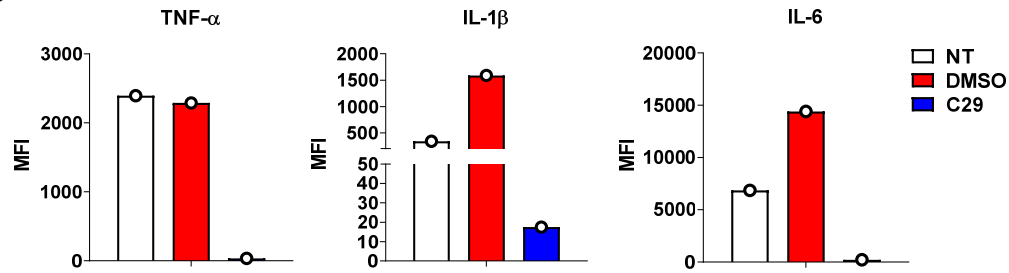**C**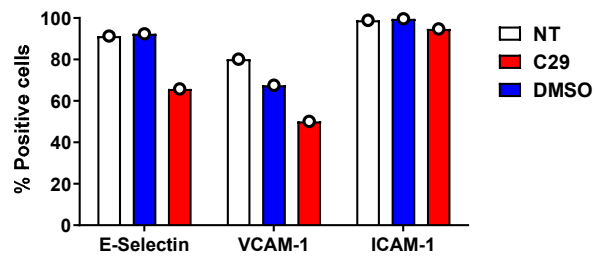

Supplement: S8 Fig — (A) PBMCs from a healthy donor (n = 1, one donor) were mock-treated with C29 (100 μM), vehicle control DMSO and PAM3CSK4 (PAM3, 600 ng/mL) for 6h, 24h and 48h. Percentage of viable PBMCs was measured by flow cytometry. (B and C) PBMCs from a healthy donor (n = 1, one donor) were mock-treated with C29 (100 μM) or vehicle control DMSO for 2h prior exposure to PAM3CSK4 (PAM3, 600 ng/mL) for 6h in the presence of Brefeldin-A. (B) Intracellular accumulation of TNF-α, IL-1β and IL-6 in the live monocyte fraction within the PBMCs was measured by flow cytometry. (C) HUVEC were mock-treated with C29 (100 μM) or vehicle control DMSO for 6h. Surface expression of E-selectin, VCAM-1 and ICAM-1 on HUVEC was determined by flow cytometry. (PDF) [file ppat.1010499.s008.pdf]

A

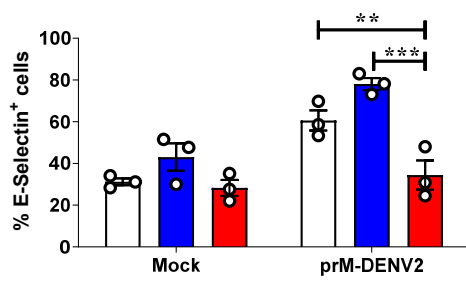

B

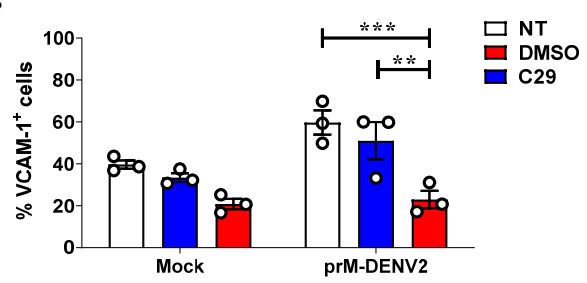

C

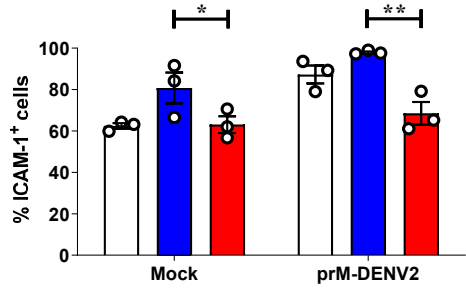

D

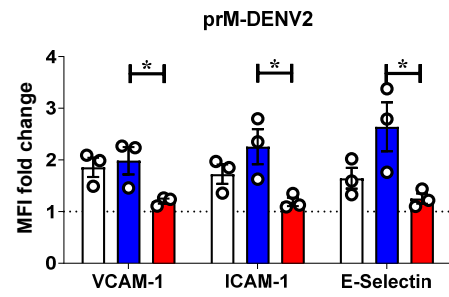

Supplement: S9 Fig — PBMCs from healthy donors (n = 3, three different donors) were mock-treated with C29 or DMSO (vehicle) for 2h prior exposure to prM-DENV2 (MOG 300) or mock treatment for 6h. Cell-free supernatants were collected and used to stimulate HUVEC. Surface expression of E-selectin, VCAM-1 and ICAM-1 on HUVEC was determined by flow cytometry and represented as (A-C) percentage of adhesion molecule positive cells and (E) MFI fold change to non-treated control. P values were obtained by unpaired one-tailed t-test (*P<0.05; ** P<0.01; ***P<0.001). (PDF) [file ppat.1010499.s009.pdf]

A

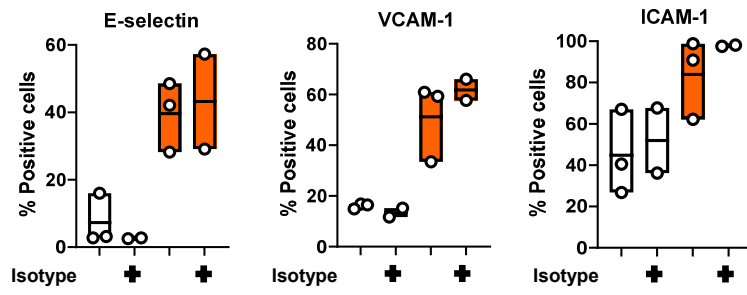

B

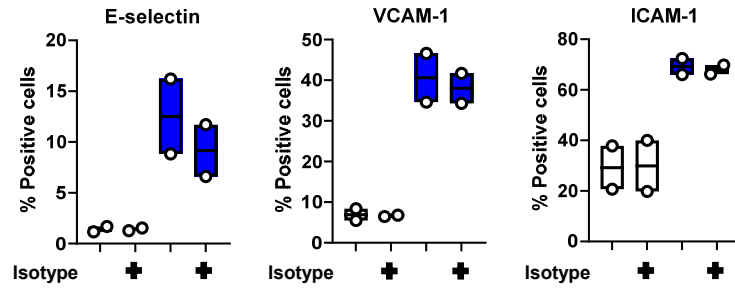

Supplement: S10 Fig — HUVEC were incubated for 6h with cell-free supernatants from PBMCs (n = 2–3, two to three different donors) (mock)-exposed for (A) 6h and (B) 48h to prM-DENV2 at an MOG of 300 in the presence or absence of TLR2 isotype control antibody (5 μg/mL). Surface expression of E-selectin, VCAM-1 and ICAM-1was determined by flow cytometry and represented as percentage of positive cells and MFI. Orange: 6h, Blue: 48h. (PDF) [file ppat.1010499.s010.pdf]

**A**

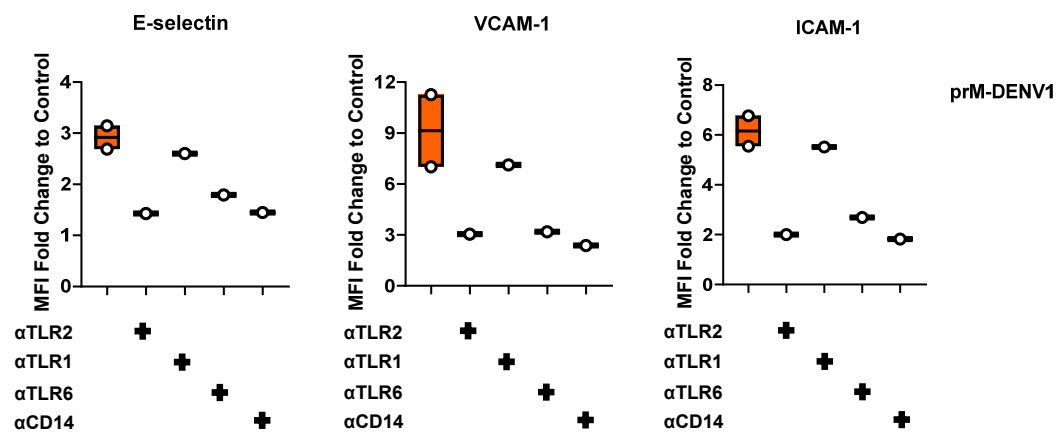

**B**

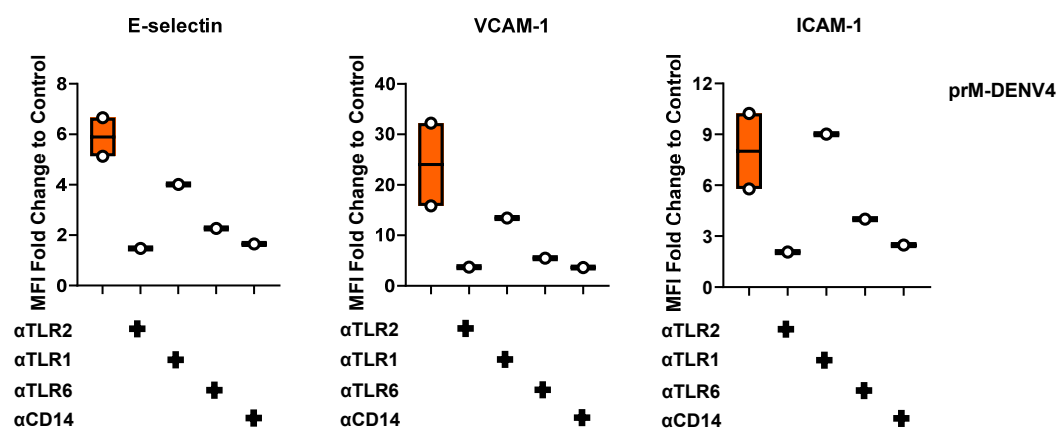

Supplement: S11 Fig — HUVEC were incubated for 6h with cell-free supernatants from PBMCs (n = 2, two different donors) exposed for 6h to (A) prM-DENV1 and (B) prM-DENV4 at an MOG of 300 in the presence or absence of TLR2 isotype control antibody (5 μg/mL). Surface expression of E-selectin, VCAM-1 and ICAM-1was determined by flow cytometry and represented as percentage of positive cells and MFI. (PDF) [file ppat.1010499.s011.pdf]

A

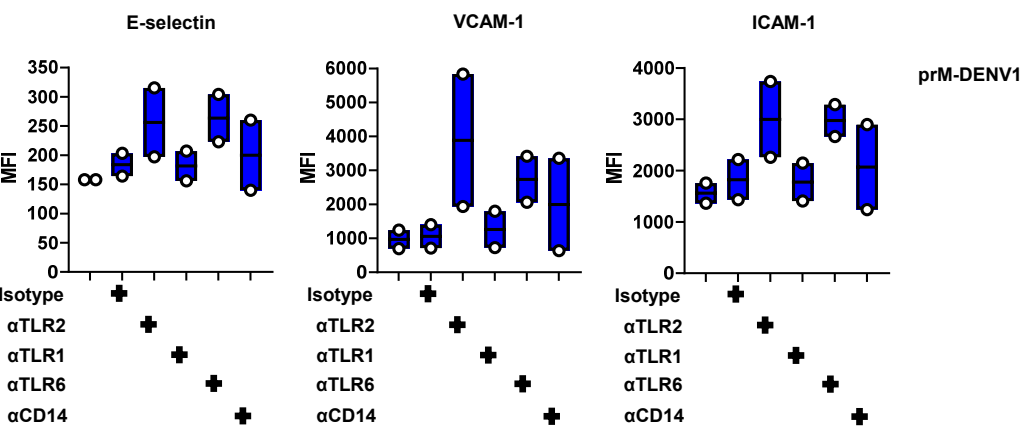

B

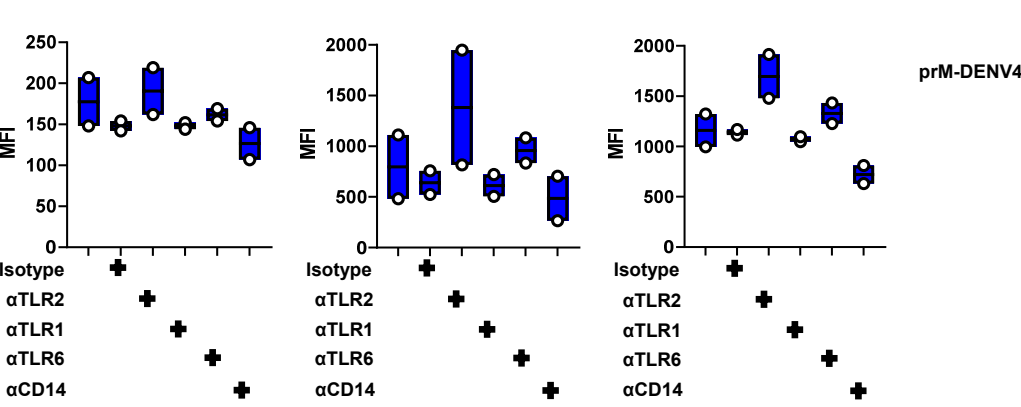

Supplement: S12 Fig — HUVEC were incubated for 6h with cell-free supernatants from PBMCs (n = 2, two different donors) exposed for 48h to (A) prM-DENV1 and (B) prM-DENV4 at an MOG of 300 in the presence or absence of TLR2 isotype control antibody (5 μg/mL). Surface expression of E-selectin, VCAM-1 and ICAM-1was determined by flow cytometry and represented as percentage of positive cells and MFI. (PDF) [file ppat.1010499.s012.pdf]

A

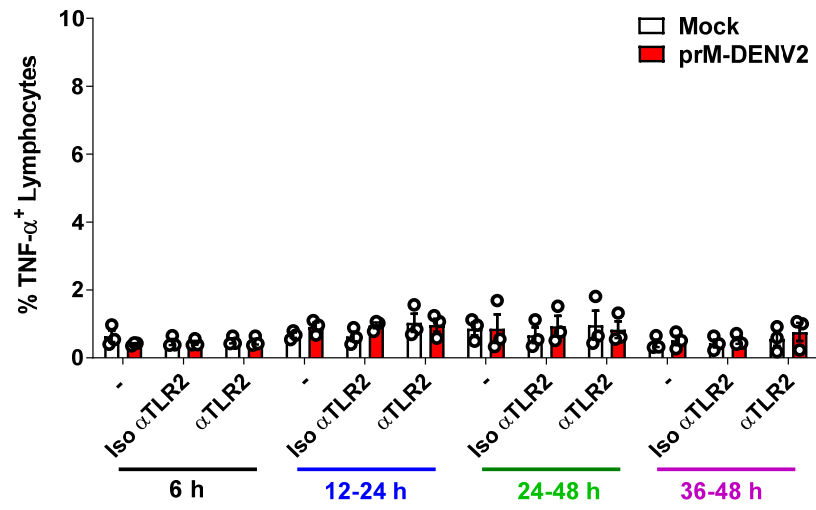

B

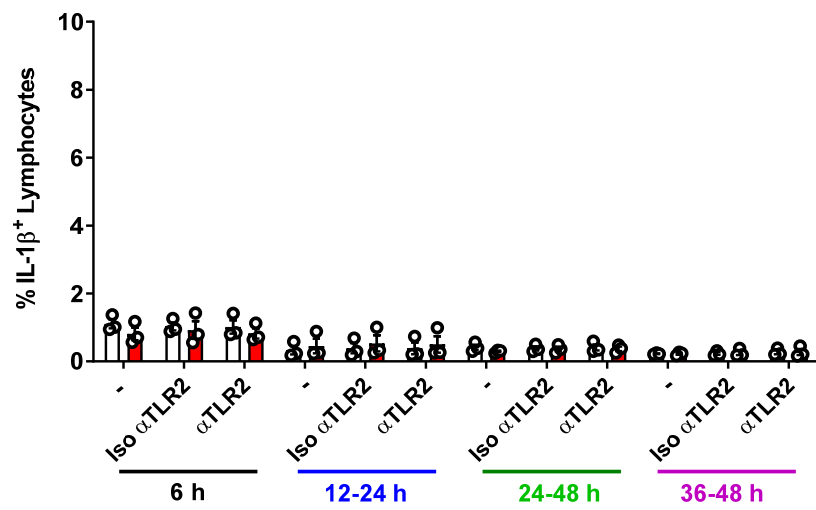

Supplement: S13 Fig — hPBMCs (n = 3, three different donors) were treated with anti-TLR2 or TLR2 isotype control antibody (5 μg/mL) for 2h prior exposure to prM-DENV2 (MOG 300) for 6h, 24h and 48h in the presence of Brefeldin-A. The intracellular accumulation of (A) TNF-α and (B) IL-1β was measured in lymphocytes (within PBMCs) by flow cytometry. Bar represents the mean ± SEM. (PDF) [file ppat.1010499.s013.pdf]

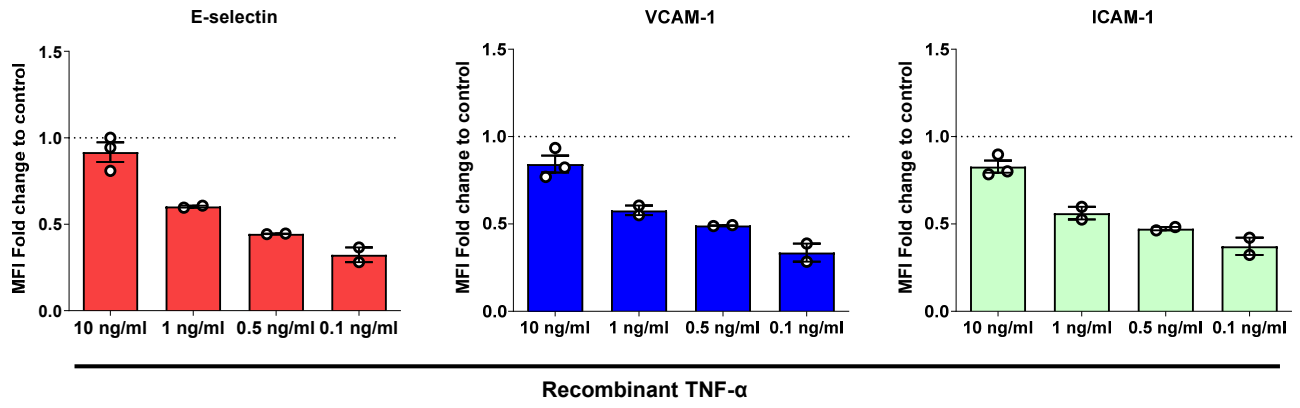

Supplement: S14 Fig — Different concentrations of recombinant TNF-α (rTNF-α, 0.1–10 ng/ml) were incubated in the presence or absence of anti-TNF-α antibody (4 μg/ml) for 1 hour. HUVEC were then stimulated with the rTNF-α/ anti-TNF-α preparations for 6h and the surface expression of E-selectin, VCAM-1 and ICAM-1 was determined by flow cytometry and showed as MFI normalized to relative mock values. Bar represents the mean ± SEM. (PDF) [file ppat.1010499.s014.pdf]
