# Supplementary material for: TLR2 axis on peripheral blood mononuclear cells regulates inflammatory responses to non-infectious immature dengue virus particles
Source: PLoS Pathog. 2022 Oct 14;18(10):e1010499. doi: 10.1371/journal.ppat.1010499 (PMC9605289; doi:10.1371/journal.ppat.1010499)
Supplement: Attachment — Submitted filename: Aguilar Briseno Response to the reviewers.pdf [file ppat.1010499.s015.pdf]

Response to Reviewers:

We thank all three reviewers for their overall very enthusiastic response to our study. We found their feedback very constructive and used it to further improve the manuscript. Here below, we have detailed the responses to each of the reviewers' comments and/or suggestions and included the requested essential data into the manuscript.

Reviewer #1

Overall summary

Dengue (caused by dengue virus DENV) is a major clinical problem without licensed vaccines nor antivirals and so understanding host-virus interactions with regard sensing and inflammation is important and my help design novel safe and effective interventions.

Aguilar-Briseño et al., here describe a follow up study from their 2020 paper linking investigating link between TLR2 DENV sensing and pathogenesis. In their previous study, immature particles were not assessed, which is where is the main novelty of this new work lies. Immature, poorly processed virions are commonly found in vivo and their biological role is poorly understood. Their work across both papers highlights the important but complex role of TLR2 sensing during DENV infection in that acute or chronic stimulation results in distinct outcomes.

Aguilar-Briseño et al use a two-cell model of DENV host interactions looking at DENV challenge of PBMCs or pure cell populations followed by incubation of conditioned media with primary vascular cells, which are heavily involved in DENV pathogenesis. To assess the contribution of immature particles, they produce fully immature virions by infection of furin-defieicnt LoVo cell lines. Additional systems looked at include various HEK reporter lines. Using these approaches, the team show immature virions are sensed by PBMCs and monocytes (and HEK cells) in a TLR2(and coreceptor TLR6/CD14)-dependent manner, which results in production of secretable factors that can activate endothelial cells. Interestingly, prolonged TLR2 activation/DENV particle incubation results in enhanced inflammatory mediator production and vascular activation, possibly linked to TNFa.

This work forms the basis of further studies exploring production of DENV immature virions and kinetics of host immune response during primary and secondary infections. It still remains uncertain whether TLR2 sensing is protective or pathogenic across the spectrum of DENV infection in people.

In general, this is a very well-written and produced study, building on - and extending - solid previous work in a scientifically and clinical relevant arena. This reviewer appreciated the ease of presentation, inclusion of various additional controls and mechanistic focus. The stats appear to be carried out appropriately. The study could have benefited from greater numbers of PBMC donors per experiment, genetic KO models of TLR2 in primary monocytes. However, where appropriate, statistical significance and magnitude of change in primary cells is clear, and combination of neutralizing antibodies and HEK cell reporters allowed mechanistic insight. I have several minor points that would help aid in interpretation of the work.

Response:

We appreciate the reviewer's comments on the high quality and comprehensiveness of the study.

Major issues:

One significant concern is the choice of controls across the experiments and the worth of additional variables in interpretation of their results. **1.-** i) Were the prMDENV preparations purified by ultracentrifugation or are they crude? How would this affect results? Ii) Would additional controls such as media from uninfected LoVo cells or from heat-treated prmDENV from LoVo be helpful?

Response:

We agree with the reviewer on the importance of the right controls and the worth of additional variables in data interpretation. With regard of the reviewer concerns, crude preparations of prM-DENV infected cells can contain inflammatory mediators or soluble cellular factors that could potentially influence our results. To test for that, we have used both purified (by ultracentrifugation) and crude clarified supernatants from prM DENV infected cells. Purified prM-DENV2 virions activated NF-κB in a similar fashion as crude supernatant-containing virions demonstrating that molecular patterns present on the virions rather than a soluble factor are responsible for the activation. We have included in Supplementary Figure 4 (panel B) a comparison on the ability of pure and crude preparations of prM-DENV2 to activate NF-κB utilizing HEK-Blue hTLR2 reporter cells.

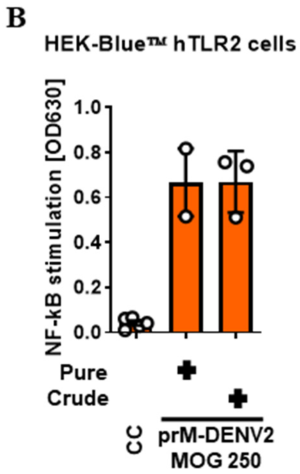

Panel B, Supplementary Figure 4. (**B**) HEK-Blue hTLR2 cells were (mock)-treated with pure (n=2) or crude (n=3) preparations of prM-DENV2 (MOG 250) for 24h. NF-κB stimulation was assessed by QUANTI-Blue, OD values show the induction of NF-κB. Data represents the mean ± SEM. CC: cellular control.

***Adjusted Results**:*

Purified prM-DENV2 activated NF-κB to the same extent demonstrating that molecular patterns present on the surface of the virus rather than a soluble factor are responsible for this activation.

2.- Iii) Furthermore, use of standard mature infectious DENV in some experiments would be useful to compare. Can the authors comment on the merits of these additional controls.

Response:

We agree with the reviewer that the inclusion of standard mature infectious DENV in some experiments would be useful for comparison. As we previously uncovered the role of TLR2 on sensing infectious DENV particles (PMID 32576819) we aimed in this manuscript to elucidate the role of non-infectious immature DENV particles in systemic inflammation. When the experiments on Figure 1 were conducted, infectious std DENV2 preparations were also tested in parallel. Our data (see below) show that infectious std DENV2 induces a relatively late onset of inflammatory responses. These data are in agreement with that of our previously published manuscript (PMID 32576819) in which we have characterized these responses in detail. Altogether our data show that, unlike std DENV2, prM-DENV2 induce a rapid onset of inflammatory mediators in PBMCs which in turn activate endothelial cells. As requested by the reviewer, we have included these data in figure 1 (panel C and D, respectively).

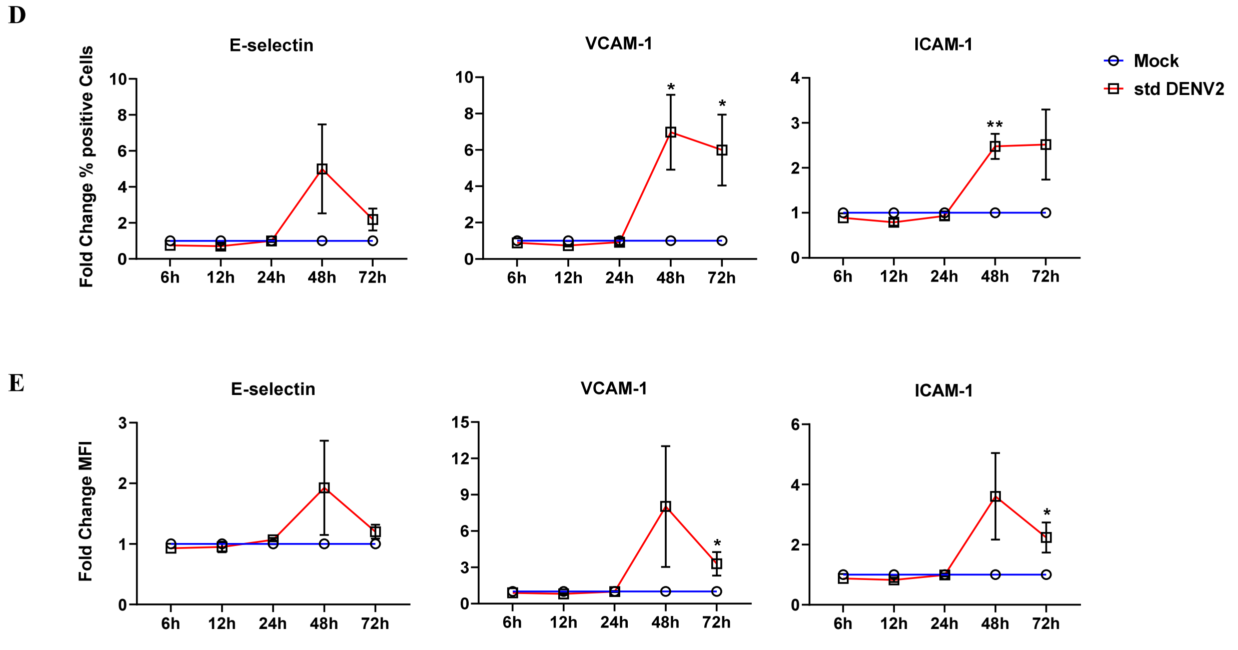

Panel D and E, Figure 1. HUVEC were incubated with cell-free supernatants harvested at indicated time points from hPBMCs (n=3, three different donors) infected to std DENV2 at an MOI of 10 or mock treatment. Surface expression of E-selectin, VCAM-1 and ICAM-1 on HUVEC was determined by flow cytometry and represented as (**D**) percentage of positive cells and (**E**) MFI normalized to relative mock values. Data represents the mean ± SEM. P values were obtained by unpaired one-tailed t test (*P<0.05; **P<0.01).

Adjusted Results:

Supernatants from PBMCs infected at a multiplicity of infection (MOI) of 10 with standard (std) DENV2 were used as a comparison.

In contrast, and as previously observed [18], std DENV2 induced an onset of soluble inflammatory responses that peaked at 48h and start resolving at 72h (Fig. 1 D, E). Importantly, and in line with our previous study [29], prM-DENV2 was virtually non-infectious in PBMCs, as evidenced by the absence of DENV-E positive cells and lack of infectious virus in PBMCs' supernatants even after 72 hours of infection, which in theory corresponds to 3 replication cycles (Supplementary Fig. 3A including std DENV2 and UV-prM-DENV2 as a positive and negative controls, respectively). In agreement with our surrogate assay, std DENV2 but not UV- prM-DENV2, UV-std DENV2 or prM-DENV2 was infectious on HUVEC as evidenced for the presence of viral antigens (E and NS3, Supplementary Fig. 3B, C). This implies that the observed kinetics of innate responses induced by prM-DENV2 are not modulated by virus replication. Altogether, our data show that prM-DENV2 can induce a rapid onset of inflammatory mediators in PBMCs, which in turn can activate endothelial cells.

Minor issues:

4.- Title: remove reference to "circulating" as this refers to in vivo situation, which was not assessed in this study. Indeed, the short title is more accurate in my opinion.

Response:
We have updated the title accordingly.

TLR2 axis on peripheral blood mononuclear cells regulates inflammatory responses to non-infectious immature dengue virus particles

5.- Update author summary - reads very similar to abstract, which is not the object of author summary.

Response:
We have updated the author summary accordingly.

We have previously demonstrated that Toll-like receptor 2 (TLR2), present on the surface of human monocytes, can sense DENV infection leading to the production of soluble inflammatory mediators and the activation of the endothelium. Interestingly, a large proportion of DENV particles released from the infected cells are not readily infectious because they did not complete the maturation process. Here we aimed to elucidate if and how these non-infectious, immature DENV particles contribute to systemic inflammation. We evaluated if monocytes sense immature DENV and found that sensing of immature DENV induced early inflammatory responses in PBMCs. Subsequently, by pharmacological inhibition of TLR2 and pathways downstream we demonstrated that TLR2 and CD14 drives the early production inflammatory mediators and

endothelial activation. Importantly however, prolonged inhibition of TLR2 induced a second wave of TNF-α release and the subsequent activation of the endothelium. Taken together, our study showed that although virtually noninfectious, immature DENV particles can contribute to inflammatory responses and that TLR2 play an important role in their initiation and resolution. We propose that the maturation status of DENV in the human host can influence the extent and kinetics of the inflammatory responses during DENV infection.

6.- Intro:
Spell out RIGI/MDA5
Add in DENV RNA genome having positive sense polarity

Response:
We have updated the text accordingly.:

To date, several PRRs have been implicated in sensing of DENV infection. For instance, endosomal TLR3 and TLR7, and cytosolic retinoic acid-inducible gene I (RIG-I) and melanoma differentiation-associated protein 5 (MDA5) have been shown to sense the DENV RNA early in the infection, thereby leading to the production of type I interferons

DENV is an enveloped virus with a single-stranded RNA genome of positive polarity belonging to the family Flaviviridae.

7.- Methods:
Cells section: add that LoVo cells lack furin in the first section listing LoVo (as well as in Viruses section)
Viruses section: is there meant to be a reference for production of prM-DENV in LoVo cells?
PMBCs: state what size well plate was used for your PBMC experiments

Response:
We have updated the text accordingly:

Human adenocarcinoma active furin-deficient LoVo cells were cultured in Ham's medium (Invitrogen) supplemented with 20% FBS.

prM-DENV1, prM-DENV2, and prM-DENV4, were produced in LoVo cells as described [28,30].

PBMCs (1x106 cell/mL, 48-well plate) or HEKs (5X104 cells/mL, 96-well plate) cells were treated with 5 µg/mL or 15 µg/mL(...)

8.- Were the same 3 donors used throughout?

Response:

For all the experiments presented in figures 1, 4, 5, panel C from figure 6, and their respective supplementary figures, the same 3 donors were used. Experiments presented in figure 7 and its respective supplementary figures, were done in a set of 3 different donors.

9.- When prm denv added, is it ever removed? What is its stability? Have the authors tried adding, removal and washing out the virions? Are the tlr2 inhibitors in the conditioned media when added the vascular cells? Is it possible this could affect endothelial cell biology?

Response:

For this study, we did not removed/washed out the prM-DENV virions. We agree with the reviewer that by removing the inoculum and washing the cells we would minimize the carryover of the virus and pre-treatment antibodies. We would like to point out however, that multiple washing steps especially shortly after one treatment are stressful to primary cells such as PBMCs, and by compromising their viability mask the treatment-specific immune responses. Therefore, instead, to ensure that the carryover of the virus was not the confounding factor in activation of the HUVEC, we assessed the responses of HUVEC at 6hpi (Supplementary Figure 1). Furthermore, in order to minimize the effect of the blocking antibodies on the TLR2 in HUVEC we made sure that their concentrations following treatments in PBMCs and addition of supernatants to HUVEC did not exert inhibition of NF-κB activity in the HEK-Blue™ hTLR2 systems. Specifically, prior treatment, the PBMCs are blocked with 5 µg/mL of the respective antibody, concentration that is further diluted by 2-fold following infection. Moreover, the concentration of the carryover blocking antibodies is reduced again by half at the moment of addition to the HUVEC.

10.- Often it is not clear the number of independent experiments nor what n refers to (wells or complete experiments on different days). Can the authors update each legend documenting this. Although noted everything seems consistent and no issues found.

Response:

We have updated our figure legends accordingly.

11.- The work would have benefited from more primary cell donors as significant variation is noted. On this, as variation is biologically meaningful, is there a possibility we can look at responses in PBMCs across experiments by donor or is it likely to reflect technical differences in experiments? Similarly, is it possible and relevant if these donors have had a previous DENV infection? Was this considered in use of donors?

Response:

We understand the concern of the reviewer. It is important to mention that for all the studies performed in this manuscript all the PBMC's used in our study belonged to healthy, DENV-seronegative donors. We have included this in Material and Methods for clarification. We agree

with the reviewer that variation is biologically meaningful, especially when working with human primary cells such as PBMCs. In total, 6 different donors were used through the study. In addition, the responses observed in different experiments in which the same donors were utilized, do reflect small technical differences between the experiments. Since the same pattern of activation was observed each time, we believe that the obtained data are biologically meaningful.

Material and Methods:

Buffy coats obtained from anonymized, DENV-seronegative, Sanquin blood donors were used to isolate PBMCs after written informed consent by the donors.

12.- Results:
Dengue virions : change to DENV virions
Alter: significant (> at least 2 fold) - does significant refer to statistics or to fold change magnitude?
Change the word "tagged" (as in tagged as std) to "referred to herein as".
Change dengue virus to DENV where applicable throughout
Re: role of tlr1, there does seem to be a trend towards lower activation upon its neutralization, although perhaps not statistically or biologically significant

Response:

To improve readability, we have updated the text accordingly. With regard of the reviewer observation on the trend towards lower activation upon TLR1 neutralization, the modest decrease in activation observed in some data is not statistically significant.

To test the ability of immature DENV virions to induce inflammatory responses in PBMCs that contribute to the vascular activation, we have used the previously established model in endothelial cells (EC).

Notably, the supernatants of prM-DENV2-challenged PBMCs induced the activation of EC as evidenced by a significant increase in the percentage of positive cells (Fig. 1B and Supplementary Fig 2A) and MFI (Fig. 1C Supplementary Fig 2B).

In that study, we used DENV preparations produced in human or mosquito cells referred to herein as standard (std).

Immature DENV particles trigger NF-κB activation in a TLR2/6 and CD14 dependent manner.

Hence, we hypothesized that the TLR2 axis could also sense immature DENV particles.

13.- Discussion:
What is sensing prmDENV immature in tlr2 block? Is there an additional sensor, possibly RNA or not, operating here during your setup?

What happens to the immature DENV upon tlr2 sensing? Are the particles internalised and removed?

Response:

We appreciate the thoughts of the reviewer on the sensing of immature DENV during TLR2 block and on the role that other pattern recognition receptors could play in our experimental set ups. We have extended in our discussion one of paragraphs already touching on the role of differential PRRs engagement in our experimental set up. In addition, we have included a discussion on the fate of immature DENV upon TLR2 sensing.

***Adjusted Discussion**:*

Immature dengue particles have been previously shown to contribute to the innate immune responses by triggering potent type I IFN responses [31]. In that study, Décembre et al., co-cultured pDCs isolated from blood with immature DENV-producing cells and observed an increase in the production of IFN-α by pDCs. Here, however, we could not detect IFN-α production in response to prM-DENV. The different cytokine responses observed in our study and that of Décembre's may be attributed to the different experimental approaches employed, enriched DC cultures vs PBMCs, in which pDCs represent less than 1% of all cells. It is worth mentioning that using the same infection model as here, we did detect IFN-type I responses in response to standard and infectious DENV preparation[18]. It is thus tempting to speculate that the noted difference is driven by the very inefficient entry, and infection of the immature DENV in PBMCs vs in pDCs enriched population, as consequence, the differential engagement of intracellular pattern recognition receptors sensing viral RNA and replication intermediates e.g., TLR3, TLR7, TLR8, RIG-I and MDA5 [13–15]. It has been previously shown that immature DENV virus can enter myeloid dendritic cells via DC-SIGN and initiate low grade infection [30]. Whether prM-DENV entry and RNA-sensing is also required for pDCs remains elusive. Also, in our PBMCs model, single TLR/CD14 treatments were studied. Therefore during the presence of TLR2 block, other TLR2 co-receptors such as CD14 and CD36 that have been shown to induce signaling independent of TLR engagement [59,60], may be sensing prM-DENV. Alternatively, C-type lectins present on myeloid cells could also play a role in the sensing [61]. Further studies are required to evaluate the differential engagement and/or crosstalk of these PRR's on PBMCs upon exposure to immature DENV in the presence of TLR2 block.

DENV is known to hijack CME to gain access to the internal compartments of host cells [20]. In addition, TLR2 ligands LTA, FSL-1 and PAM3CSK4 are internalized via CME in which TLR2 co-receptors: CD14 and CD36 play a very important role [38,40]. In the current study, we questioned if the sensing of immature DENV particles occurred at the plasma membrane and/or in an endosomal compartment. We found that internalization of immature DENV virions by the TLR2/TLR6/CD14 axis via CME potentiates subsequent NF-κB activation. These data are in line with previous findings by us and others [18,38]. However, the fate of immature DENV virions following internalization via CME is unknown. Triantafilou et al., showed that TLR2, CD14 and CD36 reside in lipid rafts, following ligand binding TLR1 and TLR6 are recruited to lipid rafts and TLR2 clusters are targeted to the Golgi apparatus. Localization and targeting inside the cell are

ligand-specific, being trafficking independent of signaling [62]. Subsequent studies should elucidate the fate of immature DENV following sensing via TLR2/TLR6/CD14 and explore the role of the TLR2/TLR6 co-receptor CD36 on signaling, trafficking and internalization.

Reviewer #2

Overall summary

In this well developed manuscript, Aguilar-Briseño and colleagues demonstrate that TLR2 in PBMCs mediates an inflammatory response to immature DENV particles. Using DENV with a significant amount of prM on its surface (prM-DENV), they show that TLR2 signaling is induced in PBMCs exposed to this virus, though not directly infected. Through pharmacological inhibition of distinct steps in the pathway, they further show that NFkB signaling and downstream cytokine leads to endothelial cell responses to infection. Finally, they nicely show that these responses are downregulated through TLR2 shedding. Overall, the manuscript is well written and understandable, the figures are nicely described and the diagrams for experiments and the model are very appreciated, and the experiments are logical.

Response:

We thank the reviewer for their assessment and positive comments in our manuscript.

Major issues:

1.- The authors primary motivation for the manuscript is the detection of prM-DENV via TLR2, which they demonstrate in Figure 1. They do not compare this response to a response from DENV infection, with less prM or with any other distribution of prM. Because the authors spend significant parts of the introduction and a full paragraph of the discussion describing the viral system, it would benefit the manuscript to have this comparison within the manuscript.

Response:

We agree with the reviewer. When the experiments for Figure 1 were conducted, infectious std DENV2 preparations were also tested in parallel. Our data (see below) show that infectious std DENV2 induces a late onset of inflammatory responses. These data are in agreement with that of our previously published manuscript (PMID 32576819) in which we have characterized PBMCs' responses to standard (std) virus preparation. Altogether our data show that, unlike std DENV2, prM-DENV2 induce a rapid onset of inflammatory mediators in PBMCs which in turn activate endothelial cells. As requested, we have included these data in figure 1 (panel C and D, respectively).

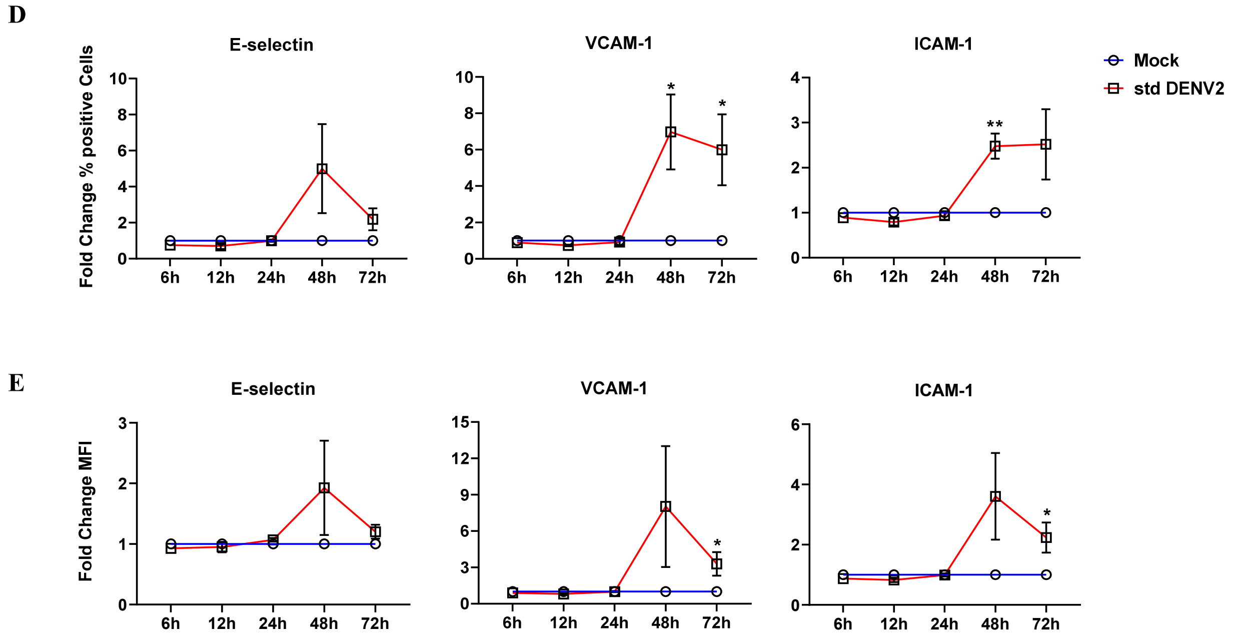

Panel D and E. Figure 1. HUVEC were incubated with cell-free supernatants harvested at indicated time points from hPBMCs (n=3, three different donors) infected to std DENV2 at an MOI of 10 or mock treatment. Surface expression of E-selectin, VCAM-1 and ICAM-1 on HUVEC was determined by flow cytometry and represented as (D) percentage of positive cells and (E) MFI normalized to relative mock values. Data represents the mean ± SEM. P values were obtained by unpaired one-tailed t test (*P<0.05; **P<0.01).

Adjusted Results:

Supernatants from PBMCs infected at a multiplicity of infection (MOI) of 10 with standard (std) DENV2 were used as a comparison.

In contrast, and as previously observed [18], std DENV2 induced an onset of soluble inflammatory responses that peaked at 48h and start resolving at 72h (Fig. 1 D, E). Importantly, and in line with our previous study [29], prM-DENV2 was virtually non-infectious in PBMCs, as evidenced by the absence of DENV-E positive cells and lack of infectious virus in PBMCs' supernatants even after 72 hours of infection, which in theory corresponds to 3 replication cycles (Supplementary Fig. 3A including std DENV2 and UV-prM-DENV2 as a positive and negative controls, respectively). In agreement with our surrogate assay, std DENV2 but not UV- prM-DENV2, UV-std DENV2 or prM-DENV2 was infectious on HUVEC as evidenced for the presence of viral antigens (E and NS3, Supplementary Fig. 3B, C). This implies that the observed kinetics of innate responses induced by prM-DENV2 are not modulated by virus replication. Altogether, our data show that prM-DENV2 can induce a rapid onset of inflammatory mediators in PBMCs, which in turn can activate endothelial cells.

2.- Their data regarding TLR2 shedding shows that samples prior to 48 hpi largely maintain TLR2 (no detectable sTLR2). Do the authors envision that the shedding of TLR2 is gradual? Additional timepoints between 24h and 48h would be informative.

Response:

We agree with the reviewer that the inclusion of timepoints between 24h and 48h would provide valuable information regarding TLR2 shedding. The new 30h data shows that TLR2 shedding start occurring around this timepoint. This observation further validates that TLR2 shedding occurs gradually during prM-DENV2 virus infection. As the reviewer requested, we have included a 30h timepoint on Figure 5, panel C.

C

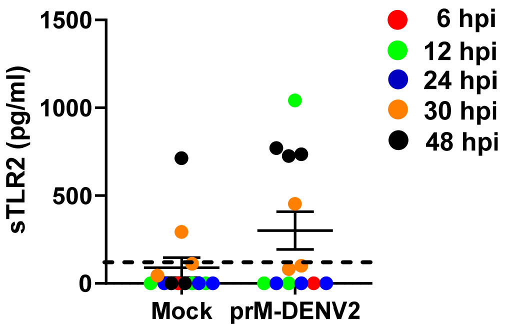

Panel C, Figure 1. PBMCs from healthy donors (n=3, three different donors) were exposed to prM-DENV2 at MOG 300 for 6h, 12h, 24h, 30h and 48h. (**C**) Concentration of sTLR2 in the supernatants exposed to prM-DENV2 determined by ELISA. Error bars represent mean± SEM

3.- It is surprising that the authors find little interferon in their samples, despite clear prM-DENV detection by the PBMCs. They attribute this to differences in the experimental systems (though their cultures also have DCs) or differences in the virus. Their suggestion that efficient infection leads to detection and IFN production is testable within their system, and would significantly benefit this statement.

Response:

We understand the query of the reviewer finding surprising the lack of type I IFN production in our experimental set up, despite the clear detection of immature DENV by PBMCs. Important to mention is that engagement of TLR2 does not always lead to the production of IFNs, nature of the ligand and origin of the signaling (plasma membrane vs endosomal compartment) influence IFN production (PMID 19801985). We previously demonstrated (PMID 32576819) that DENV infection induce a late onset of inflammatory responses in PBMCs which in turn activate the endothelium. Those responses were characterized by the production of soluble inflammatory mediators such as

IP- IFN-α2, IFN-β and IFN-λ1 and were dependent on viral replication as UV-Inactivated std DENV2 failed to induce the production of these inflammatory mediators. In our current study, prM-DENV2 induced a TLR2-dependent early onset of inflammatory responses characterized by the production of TNF, IL-1β and IL-6. Important to mention, and in line with our previous study, std DENV only induced inflammatory responses late in the course of infection (48h) (Figure 1). In addition, our highly sensitive surrogate infection assay on highly permissive BHK-15 cells (Supplementary Figure 3) demonstrated that supernatants from 48h prM-DENV2-exposed PBMCs lack infectious virus, corroborating previous findings that prM-DENV is virtually non-infectious. Our previous published data and our current findings showing the differential responses triggered by prM-DENV and std DENV, validate our premise that efficient infection detection led to production of IFN.

Minor issues:

4.- Supplementary Figure 7 (or portions of it) seem rather important to the manuscript and it may benefit the paper to have some of these data in the main body of the text.

Response:

We thank the reviewer for their suggestion. As requested, we have removed panel G from Supplementary Figure 7 and included these data set in Figure 4 (Panel F, see below). As we mentioned in the Results section, the TLR2 agonist PAM3 served as a reference to compare the pathways involved in the production of cytokines downstream TLR2. Ergo, this comparison should be part of the main panel sets of Figure 4.

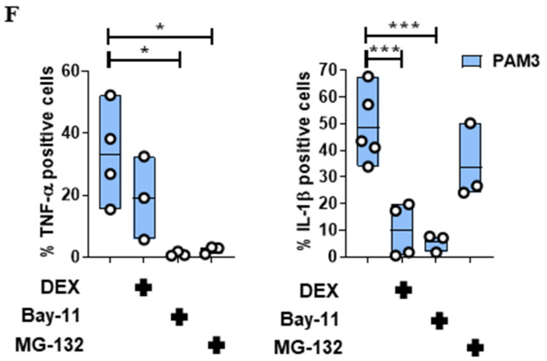

Panel F, Figure 4. PBMCs from healthy donors (n=3-5, three to five different donors) were mock-treated with Dexamethasone (DEX, 10µM), Bay-11 (5µM) and MG-132 (9.5 µg/mL) for 2 hours prior exposure to PAM3CSK4 (PAM3, 600 ng/mL) for 12h in the presence of Brefeldin-A. (**F**) Intracellular accumulation of TNF-α and IL-1β in live monocytes. Bar represents the mean ± SEM. P values were obtained by unpaired one-tailed t test. (*P<0.05; ***P<0.001).

Adjusted results:

However, in response to PAM3, DEX inhibited the accumulation of only IL-1β while no effect on TNF-α was observed (Fig. 4F). This highlights the diversity and agonist-specificity of signaling pathways downstream of TLR2 [46]. Bay-11, which selectively targets the NF-κB signaling pathway, significantly abrogated the accumulation of both cytokines induced by prM-DENV2 and PAM3 (Fig. 4E, F).

5.- The difference in the response at 6h and 48h (Figure 5) is interesting and the authors nicely provide explanations for this difference. Their data would seem to indicate that blockage of TLR2/TLR6/CD14 enhances this late response. Are these data statistically significant?

Response:

As the reviewer pointed out, our late responses data seem to indicate that blockage of TLR2/TLR6/CD14 would enhance these responses. However, we refrained to do statistical analysis of these as initially we only had data from two donors. In addition, the usage of isotype control antibodies (supplementary figure 10) further validated the response observed at 48hpi. We have now included data from 3 additional donors in Figure 5 (Panel C, 48hpi) for mock, prM-DENV2 and prM-DENV2 under TLR2 block condition, respectively. Our data show that TLR2 block significantly increased the cell frequencies of the adhesion molecules E-selectin, VCAM-1 and ICAM-1.

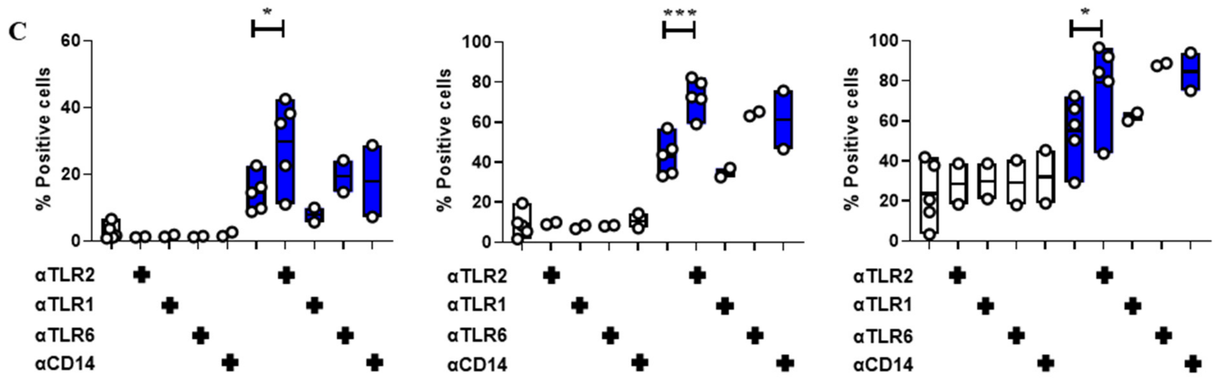

Panel C, Figure 5. HUVEC were incubated for 6h with cell-free supernatants of PBMCs (2 to 5 donors) (mock)-exposed for (**C**) 48h to prM-DENV2 at a MOG of 300 in the presence or absence of TLR2, TLR1, TLR6, CD14 blocking antibodies. Surface expression of E-selectin, VCAM-1 and ICAM-1 was determined by flow cytometry and represented as percentage of positive cells. P values were determined by unpaired one-tailed t-test. (*P<0.05, ***P<0.001).

Adjusted Results:

Surprisingly, however, while TLR2 block mitigated the inflammatory responses of PBMCs at 6hpi, the opposite effect was observed at 48hpi time-point. There, the presence of TLR2 block during prM-DENV2 exposure significantly increased the frequencies of the three adhesion molecules on EC (Fig. 5C), similar responses were observed when blocking TLR6 and/or CD14.

Reviewer #3

Overall summary

This study is very similar to the author's prior work which showed that TLR2 on monocytes is activated by circulating dengue virus (PMID 32576819). Previously the authors had not specified whether mature or immature virions can trigger this pathway. Here they show TLR activation of PBMCs by immature DENV and characterize the down-stream signaling that follows TLR2 activation, which is an already well characterized pathway. Like their previous study, they again show that monocytes are the main responders in PBMCs to stimulation by virus (here immature virus). The proportions of mature and immature virus in vivo are not known so it is not clear how consequential this distinction is for physiological function.

Response:
We thank the reviewer for their comments and suggestions.

Major issues:

1.- There is no comparison between mature and immature triggered responses so we cant know if the central claim of the study that immature virus is uniquely triggering this pathway is actually true. This is a control missing throughout the study.

Response:

We agree with the reviewer. When the experiments on Figure 1 were conducted, infectious std DENV2 preparations were also tested in parallel. Our data (see below) show that infectious std DENV2 induces an onset of inflammatory responses that peaks 48 h post infection. These data are in agreement with that of our previously published manuscript (PMID 32576819) in which we have characterized these late responses. As requested by the reviewer, we have now included these data in figure 1 (panel D and E, respectively). Altogether our data show that, unlike std DENV2, prM-DENV2 induce a rapid onset of inflammatory mediators in PBMCs which in turn activate endothelial cells.

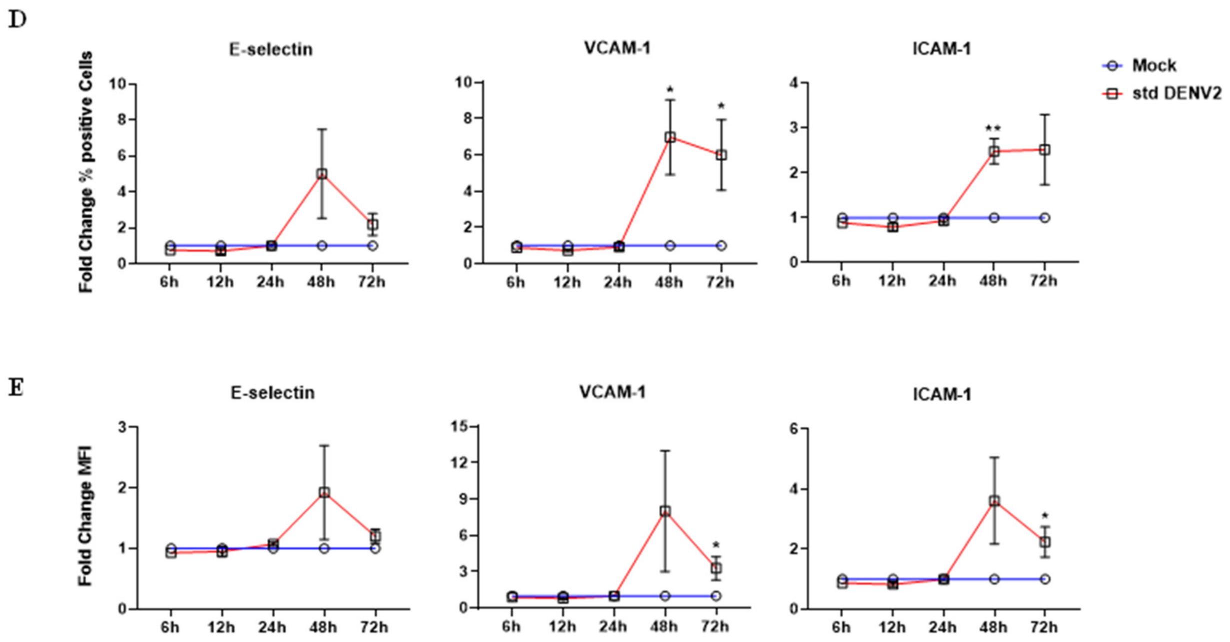

Panel D and E. Figure 1. HUVEC were incubated with cell-free supernatants harvested at indicated time points from hPBMCs (n=3, three different donors) infected to std DENV2 at an MOI of 10 or mock treatment. Surface expression of E-selectin, VCAM-1 and ICAM-1 on HUVEC was determined by flow cytometry and represented as (**D**) percentage of positive cells and (**E**) MFI normalized to relative mock values. Data represents the mean ± SEM. P values were obtained by unpaired one-tailed t test (*P<0.05; **P<0.01).

Adjusted Results:

Supernatants from PBMCs infected at a multiplicity of infection (MOI) of 10 with standard (std) DENV2 were used as a comparison.

In contrast, and as previously observed [18], std DENV2 induced an onset of soluble inflammatory responses that peaked at 48h and start resolving at 72h (Fig. 1 D, E). Importantly, and in line with our previous study [29], prM-DENV2 was virtually non-infectious in PBMCs, as evidenced by the absence of DENV-E positive cells and lack of infectious virus in PBMCs' supernatants even after 72 hours of infection, which in theory corresponds to 3 replication cycles (Supplementary Fig. 3A including std DENV2 and UV-prM-DENV2 as a positive and negative controls, respectively). In agreement with our surrogate assay, std DENV2 but not UV- prM-DENV2, UV-std DENV2 or prM-DENV2 was infectious on HUVEC as evidenced for the presence of viral antigens (E and NS3, Supplementary Fig. 3B, C). This implies that the observed kinetics of innate responses induced by prM-DENV2 are not modulated by virus replication. Altogether, our data show that prM-DENV2 can induce a rapid onset of inflammatory mediators in PBMCs, which in turn can activate endothelial cells.

2.- All of the studies presented were obtained using a culture system where supernatants are transferred from DENV-activated PBMCs to endothelial cells. There are no in vivo studies or validation that these responses occur in vivo

Response:

We understand the concern of the reviewer. However, the main focus of our manuscript is to understand the mechanisms by which immature DENV particles may contribute to inflammation. It is important to note that due to differential (low vs high) expression of TLR2 on, inter alia, human vs murine vascular endothelial cells respectively, studying the role of this PRR in dengue pathogenesis in a simple mouse model could lead to interesting but clinically irrelevant results (PMID 17986487). We therefore believe that our study forms a solid basis for further investigations elucidating the role of immature particles during natural DENV infection. Those studies, however, are beyond the scope of the present manuscript.

3.- Given the fact that it has already been shown that DENV activates TLR2 signaling, most of the results of experiments performed validating cytokine production and NFkB activation etc. are fully expected.

Response:

In our previously published data (PMID 32576819), we have characterized the innate immune responses elicited by sensing infectious std DENV via TLR2/TLR6/CD14 axis. Those TLR2-mediated responses were dependent on viral replication and involved the production of IFNs. In this study, and per this reviewer request, we have included data from infectious std DENV2 preparations. Our data, as previously discussed above, are in line with our published one. Interestingly, while std DENV2 responses occurred late in the course of infection, prM-DENV2 rather induced an early onset of inflammatory responses. Both responses have different involvement of pro-inflammatory mediators. Thus, the results of our experiments validating cytokine production and NF-κB activation may not be fully expected.

4.- I don't think the flow cytometry based assay is sensitive enough to establish that there was not virus replication. Negative-strand PCR should be used. Also, this should be done on the HUVEC cells too, not only the PBMCs.

Response:

We do believe that our surrogate infection assay on highly permissive BHK-15 cells is sensitive enough to establish if viral replication occurred on PBMCs (Panel A, Supplementary Figure 3). We would like to point out that in this assay we used PBMCs supernatants harvested at 48h after exposure of the cells to prM-DENV2. Since BHK-15 are very susceptible and permissive to DENV infection, the lack of any positive cells after 24 hpi is a strong indication of lack of productive infection in PBMCs, corroborating previous findings (PMID 19008392, PMID 20062797, PMID 24886790) that prM-DENV is indeed virtually non-infectious. We agree with the reviewer that this

analysis should have been done on HUVEC, too. Accordingly, as suggested by the reviewer, we exposed HUVEC for 24hpi, 48hpi, and 72hpi to UV- prM-DENV2, UV-std DENV2, prM-DENV2 and std DENV2. In agreement with our surrogate assay, std DENV2 but not UV- prM-DENV2, UV-std DENV2 or prM-DENV2 was infectious on HUVEC as evidenced for the presence of viral antigens (E and NS3). The data has been included as Supplementary Figure 3, panel B (staining of E protein + cells by immune fluorescent microscopy at 24 hpi) and panel C (staining of non-structural protein 3, NS3, by western blot at 24, 48 and 72hpi) and (quantification of NS3 intensity relative to the GAPDH at 24hpi, 48hpi and 72hpi by western blot). Altogether, our data demonstrate that prM-DENV2 is not capable to establish infection in PBMCs and HUVEC. We have included these data in Supplementary Figure 3, panels B and C, respectively.

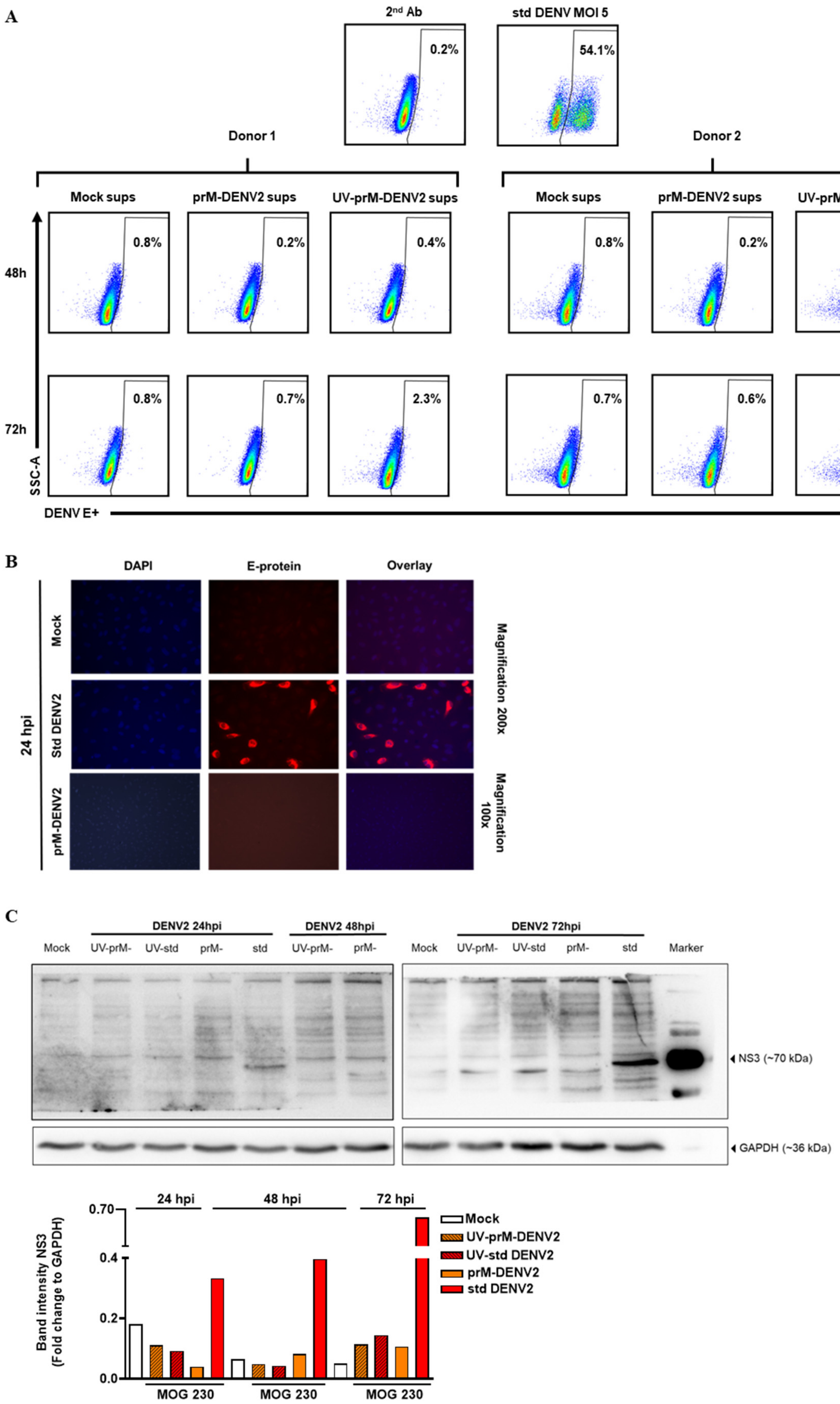
A
2nd Ab
0.2%
std DENV MOI 5
54.1%
Donor 1
Donor 2
Mock sups
prM-DENV2 sups
UV-prM-DENV2 sups
48h
0.8%
0.2%
0.4%
0.8%
0.2%
1.2%
72h
0.8%
0.7%
2.3%
0.7%
0.6%
0.5%
SSC-A
DENV E+
B
DAPI
E-protein
Overlay
24 hpi
Mock
Std DENV2
prM-DENV2
Magnification 200x
Magnification 100x
C
DENV2 24hpi
DENV2 48hpi
DENV2 72hpi
Mock
UV-prM-
UV-std
prM-
std
Marker
NS3 (~70 kDa)
GAPDH (~36 kDa)
24 hpi
48 hpi
72 hpi
Band intensity NS3 (Fold change to GAPDH)
0.70
0.4
0.2
0.0
MOG 230
Mock
UV-prM-DENV2
UV-std DENV2
prM-DENV2
std DENV2

Supplementary Figure 3. Immature DENV2 is not infectious in human PBMCs and HUVEC. (**A**) Baby Hamster Kidney cells clone 15 (BHK-15) were incubated for 24h with standard (std) DENV at an MOI of 5 (positive control) or cell-free supernatants from PBMCs (n=2, two different donors) exposed to prM-DENV2 (MOG 300), UV-prM-DENV2 (MOG 300) or mock treatment for 48h and 72h. % of DENV E+ cells was determined by flow cytometry. (**B)** HUVEC were infected with Std DENV2 (MOI 20) and prM DENV2 (MOG 10 000) for 24h, cells were then stained for flavivirus E-protein (n=1). (**C** and **D**) HUVEC were infected with prM-DENV2 and Std DENV2 and their respective UV-inactivated preparations at MOG 230 for 24h, 48h and 72 h. Detection of non-structural protein 3 (NS3) was detected by Western blot. GAPDH was used as a reference. Representative blot and Quantification of NS3 expression shown as fold change to the respective GAPDH control (n=1).

5.- Growing virus using Lovo cells to prevent maturation could be used for some studies, but this is an artificial system and may not represent what actually occurs in DENV infection.

Response:

In our study, we aimed to elucidate if and mechanistically how non-infectious immature dengue virus particles contribute to systemic inflammation. For this purpose and, as the reviewer pointed out, the immature DENV preparations produced in furin-defective LoVo cells are the right model to address our research questions. Our fundamental questions form the basis for further studies elucidating the role immature DENV particles in vivo during DENV infection. We have removed the word "circulating" from the title to avoid the confusion.

6.- Although it is suggested that TLR2 shedding correlates with results in co-cultured endothelial cells, this was not explicitly shown.

Response:

We agree that the correlation of TLR2 shedding with the extent and kinetics of inflammation was not explicitly shown. We have adjusted the wording accordingly and now state that "downregulation of the surface levels of TLR2 inter alia via shedding could contribute to controlling the extent and kinetics of inflammation during DENV infection. Further studies are needed to validate this premise".
