# Supplementary material for: TLR2 axis on peripheral blood mononuclear cells regulates inflammatory responses to non-infectious immature dengue virus particles
Source: PLoS Pathog. 2022 Oct 14;18(10):e1010499. doi: 10.1371/journal.ppat.1010499 (PMC9605289; doi:10.1371/journal.ppat.1010499)
Supplement: Attachment — Submitted filename: Aguilar Briseno Response to the reviewers.pdf [file ppat.1010499.s016.pdf]

Response to Reviewers:

Reviewer #1: The authors have addressed all of my concerns and I wish them all the best for publication and their future work.

Reviewer #2: The authors have fully and thoroughly addressed my concerns, as well as the other reviewers', and it is my opinion that the manuscript is sufficient for publication.

We thank the reviewers 1 and 2 for acknowledging the suggested improvements made and endorsing the publication of the revised manuscript. We would like to mention that "Major Issues" raised by the reviewers 1, 2 and 3 were regarding figure 1 and were largely shared. We are thus surprised that the made changes were acknowledged by all but Reviewer #3. Here below we address the remaining comments of the reviewer #3.

Reviewer #3:

<u>Major issues:</u>

Major issues remain after revision and some were even introduced with the added data. The addition of infectious virus added in response to Reviewer 3 Question 1 also does not clarify the question about the specificity to prM. Although they have added infectious DENV to Fig. 1 in panels D-E, this is separated from panels B-C and no statistical comparison was made between them that would allow the conclusion that the kinetics are different. Figure 1 legend does not clearly state which serotypes/strains were used and if they are matched for comparison between B-C. No concentrations of virus are provided in the figure legend and it is unclear if the same concentrations of virus were used in B-C versus D-E, so these cannot be directly compared without first establishing the similar inoculation was used and the only difference is prM. (Protein titration and addition of equivalent titers based on that might help). This is a major concern since the author's central conclusion of this manuscript is that immature virus activates inflammatory cascades earlier (on a different time course). I don't think this conclusion has been supported with data yet.

We have now revised Figure 1 according to the reviewer 3 suggestions and show std DENV and prM data in one graph. Importantly, the same MOG=300 was used for std and prM virus preparation to ensure fair comparison between the samples. The experimental details were stated in the Material and Methods section and now are also mentioned in figure 1 legend. Every figure legend states the respective prM/std DENV serotype utilized. Additionally, Material and Methods section states the strains utilized for each serotype. Based on the repetitive character of the findings using different virus stocks (always prepared in the same manner as detailed in method section) and different PBMC donors, we strongly believe that our data conclusively demonstrate different kinetics of inflammatory responses triggered by prM and std virus preparation. This conclusion is now also supported by statistical analysis shown in Figure 1 Panels B and C (See Figure 1B and C below).

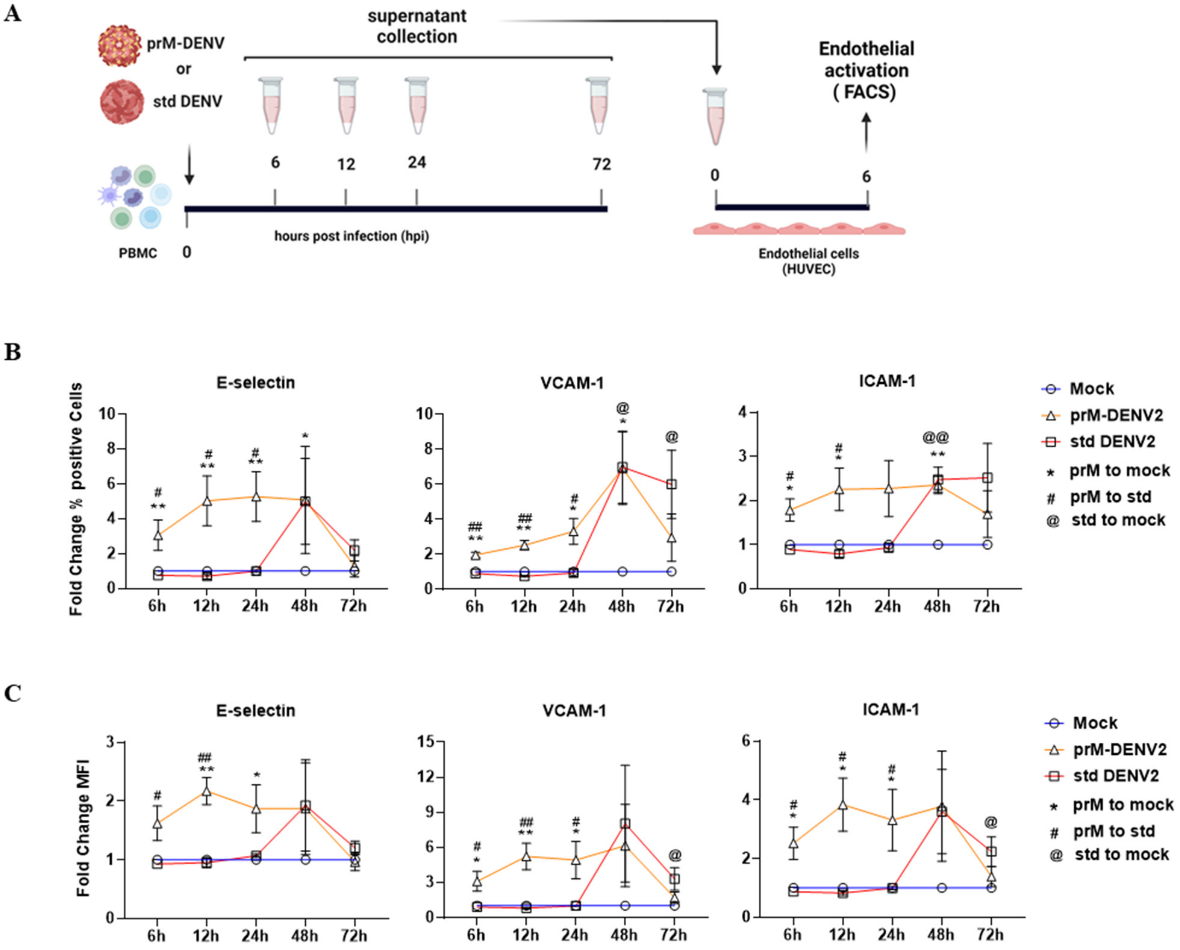

Fig. 1. prM-DENV2 induces an early onset of soluble inflammatory responses. (**A**) Experimental scheme: HUVEC were incubated with cell-free supernatants harvested at indicated time points from hPBMCs (n=3, three different donors) exposed to prM-DENV2 (MOG 300), std DENV2 (MOG300) or mock treatment. Surface expression of E-selectin, VCAM-1 and ICAM-1 on HUVEC was determined by flow cytometry and represented as (**B**) percentage of positive cells and (**C**) MFI normalized to relative mock values. Data represents the mean ± SEM. P values were obtained by unpaired one-tailed t test ($^{*/\#/@}$P<0.05; $^{**/\#\#/@@}$P<0.01).

Results:

Notably, the supernatants of prM-DENV2-challenged PBMCs induced the activation of EC as evidenced by a significant increase in the percentage of positive cells (Fig. 1B and Supplementary Fig. 2A) and MFI (Fig. 1C and Supplementary Fig 2B) of adhesion molecules compared with their respective mock-treated and std DENV2-infected conditions. Furthermore, the soluble inflammatory responses induced by prM-DENV2 were transient with a peak response between 6-24 hpi and resolution was seen between 48-72 hpi (Fig. 1B, C). In

contrast, and as previously observed [18], std DENV2 induced an onset of soluble inflammatory responses that peaked at 48h and start resolving at 72h (Fig. 1 B, C).

Material and Methods:

Standard (std) DENV1 (strain 16007), DENV2 (strain 16681) and DENV4 (strain 1036) were produced in the Aedes albopictus C6/36 cell line as described before [29]. Briefly, an 80% confluent monolayer of cells was infected at multiplicity of infection (MOI) of 0.1. Virus progeny was harvested at 72 or 168 hours post infection (hpi) depending on the serotype. prM-DENV1 (strain 16007), prM-DENV2 (strain 16681), and prM-DENV4 (strain 1036), were produced in LoVo cells as described [28,30].

Viral preparations were analyzed with respect to the infectious titer and the number of genome equivalents copies. The infectivity of immature DENV was determined by measuring the number of plaque-forming units (PFU) by plaque assay on BHK-15 cells and the number of genome-equivalent copies (GEc) by quantitative RT-PCR (RT-qPCR), as described previously [28,66]. In line with previous findings [28], the specific infectivity of prM-DENV preparations is on average 10,000 lower than that of std DENV preparation with GEC to PFU ratio of 10^6 vs std DENV2 between $5x10^1$-$5x10^2$.

PBMCs (1x106 cell/mL, 48-well plate) or HEKs (5X104 cells/mL, 96-well plate) cells were treated with 5 µg/mL or 15 µg/mL, respectively of anti-hTLR2-IgA (clone B4H2), pab-hTLR6-IgG (polyclonal), pab-hTLR1-IgG (polyclonal), anti-hCD14-IgA (2.5 µg/mL (PBMCs), clone D3B8), human IgA2 isotype control (clone T9C6), normal rat PAb IgG control blocking antibodies (InvivoGen), Dexamethasone (10µM, InvivoGen), Bay-11 (5µM, InvivoGen) or MG-132 (9.5µM, InvivoGen), C29 (100µM, MedChemExpress) for 2h followed by treatment with PAM3CSK4 (600 ng/mL (PBMCs), 50 ng/mL (HEK's), InvivoGen), prM-DENV1 (MOG 250, MOG 300), prM-DENV2 (MOG 250, MOG 300, MOG 1000), prM-DENV4 (MOG 250, MOG 300, MOG 1000) and, UV-I prM-DENV2 (MOG 300) and std DENV2 (MOG 300) for 6h, 12h, 24h, 48h and 72h.

"The authors have added LoVo cells to the manuscript methods, but they don't clearly state if they were used to grow all of the the immature virus in the text. This is very confusing. Since this aspect of the conclusions, that the response is dependent on immature virus is a central conclusion it seems important to do this experiment. If you re-read the response to Reviewer 3 Question 1, this response is not directly answering the question so it's hard to evaluate what the authors have done"

We are not sure we understand the confusion of the reviewer. All immature virus preparations were produced in LoVo cells as indicated in the Material and Methods section, just as all (std) DENV2 preparation were grown in C6/36 cells.

"The authors did not address some of the valid reviewer concerns, such as reviewer 1's request to add more human donors since there was very high variability. That comment was not addressed in the slightest and it was written that 6 donors were used, but this is misleading because there are only 3 in any given experiment."

We pity Reviewer #3 disagrees with the Reviewer #1. We have added the information requested by the reviewer #1 and substantiated the variability found in different donors to his

satisfaction: "We agree with the reviewer that variation is biologically meaningful, especially when working with human primary cells such as PBMCs. In total, 6 different donors were used through the study. In addition, the responses observed in different experiments in which the same donors were utilized, do reflect small technical differences between the experiments. Since the same pattern of activation was observed each time, we believe that the obtained data are biologically meaningful". Furthermore, every figure legend states the number of donors utilized in each experiment. We would like to highlight that data presented in Figures 1, 4, 5B and 6 was obtained with different set of donors than that of Figure 7. Data showed in Figure 5C was obtain from both set for donors. Moreover, these experiments were performed by different lab members. Despite these variables, the results show the same pattern of inflammatory responses. Consequently, we do not believe that addition of extra donors per figure would affect the overall conclusion of the study.

Ad. 4. The authors also don't validate the lack of infection by negative strand virus PCR, which is a very simple control to do and it's concerning why they are unwilling to add simple inexpensive controls.

Over the years we have used detection of infection in PBMCs with methods such us FACS (quantification of DENV-positive cells) or RT-qPCR and plaque assay (detection of genome equivalent copies or infectious particles in the cell supernatants). Using all these methods, prM particles produced in LoVo cells have proven to be virtually noninfectious in many cells and PBMC (PMID: 20062797). We use the word " virtually" because for immature DENV preparations in LoVo cells, 1 in a million particles is in fact infectious in susceptible cell monolayers (PMID: 19008392, PMID: 24886790). Consequently, MOG 300 of an average prM DENV preparation, represents an MOI of 0,0003. Furthermore, because in all our experiments the MOG is calculated based on all PBMC, not the number of monocytes, which various between 5-15% of all PBMCS depending on the donor, the effective MOI is even 10-fold lower. In fact, this is likely why we have not been successful in detecting the presence of + or - RNA in PBMC exposed to infectious (std) DENV at MOG 300 (which depending on the virus preparation represents MOI of 0.3-5). Consequently, with the specific infectivity of prM preparation being average 10.000x lower than that of the std, the qPCR-based detection of viral RNA following exposure of the cells to prM DENV although at least theoretically easy - seemed senseless.

We pity the reviewer did not acknowledge the addition of the requested WB and IF data for EC that further validate our current data (and previously published data from several groups) showing that immature virions are not capable of initiating productive infection in PBMCs and EC. In addition, we have added the information on specific infectivity of prM vs std DENV preparation in the Methods section to highlight the virtual non-infectiousness of the immature virus preparations.

Minor Issues:

Reviewer #3:

Ad. 1. The new Figure 1C is confusing. The authors should show the time course on the x-axis and use the donors as different colors indicated in the figure legend.

We have revised the Figure 1 to address the comments of all reviewers and believe the figure is now clear.

Ad. 2. I don't think it's accurate to refer to TLR2 as an "axis".

We have studied not only the contribution of TLR2 extracellular domain but with use of C29 and other inhibitors also intracellular signal transduction pathway. Therefore, the word axis captures the message better than solely TLR2.

.
